# Synuclein impairs trafficking and signaling of BDNF in a mouse model of Parkinson’s disease

**DOI:** 10.1038/s41598-017-04232-4

**Published:** 2017-06-20

**Authors:** Fang Fang, Wanlin Yang, Jazmin B. Florio, Edward Rockenstein, Brian Spencer, Xavier M. Orain, Stephanie X. Dong, Huayan Li, Xuqiao Chen, Kijung Sung, Robert A. Rissman, Eliezer Masliah, Jianqing Ding, Chengbiao Wu

**Affiliations:** 1 Department of Neurology and Institute of Neurology, Ruijin Hospital, Shanghai Jiao Tong University School of Medicine, Shanghai, China; 2 Department of Neurosciences, University of California San Diego, La Jolla, California, USA; 3 San Diego, California USA

## Abstract

Recent studies have demonstrated that hyperphosphorylation of tau protein plays a role in neuronal toxicities of α-synuclein (ASYN) in neurodegenerative disease such as familial Alzheimer’s disease (AD), dementia with Lewy bodies (DLB) and Parkinson’s disease. Using a transgenic mouse model of Parkinson’s disease (PD) that expresses GFP-ASYN driven by the PDGF-β promoter, we investigated how accumulation of ASYN impacted axonal function. We found that retrograde axonal trafficking of brain-derived neurotrophic factor (BDNF) in DIV7 cultures of E18 cortical neurons was markedly impaired at the embryonic stage, even though hyperphosphorylation of tau was not detectable in these neurons at this stage. Interestingly, we found that overexpressed ASYN interacted with dynein and induced a significant increase in the activated levels of small Rab GTPases such as Rab5 and Rab7, both key regulators of endocytic processes. Furthermore, expression of ASYN resulted in neuronal atrophy in DIV7 cortical cultures of either from E18 transgenic mouse model or from rat E18 embryos that were transiently transfected with ASYN-GFP for 72 hrs. Our studies suggest that excessive ASYN likely alters endocytic pathways leading to axonal dysfunction in embryonic cortical neurons in PD mouse models.

## Introduction

Parkinson’s disease (PD), one of the most common neurodegenerative diseases, is pathologically characterized by progressive loss of midbrain dopamine neurons and gradual development of intracellular proteinaceous aggregates termed Lewy bodies (LBs) and Lewy neurites (LNs). LBs and LNs are composed predominantly of the protein α-synuclein (ASYN)^1^. Several point mutations of the SNCA gene coding for ASYN have been identified and found to be associated with autosomal dominant forms of PD^2–4^. Duplication, triplication or overexpression of the SNCA gene has been found to induce early-onset PD^5, 6^. Genome wide association studies (GWAS) provide evidence that ASYN is also linked to sporadic PD^7^. In addition, adeno-associated viral vectors (AAV)-mediated overexpression of ASYN in rodents resulted in neurodegeneration, resembling pathological changes in PD patients^8, 9^. These findings all point to an important role played by excessive accumulation of ASYN in the pathogenesis of PD.

Although the normal function of ASYN remains to be defined, significant efforts have been made to understand the cellular processes and pathways impacted by excessive ASYN. Studies have revealed that many important cellular processes and events such as synaptic vesicle recycling, intracellular trafficking, mitochondrial energetics, lysosomal activity and autophagy etc, are all susceptible to ASYN toxicity, suggesting a multifaceted mode of neuronal toxicities by accumulation of ASYN.

ASYN significantly impacts intracellular vesicular trafficking^10, 11^. A number of Rab GTPase family members that interplay with ASYN have been identified through a large scale shRNA screening^12, 13^. These Rab proteins appear to modulate the protein level, aggregation, spreading and also toxicity of ASYN^10^. For instances, Rab8b, Rab11a, Rab13 and Slp5 all have been found to promote the clearance of ASYN inclusions and prevent ASYN-induced toxicity^12, 13^. Intriguingly, using an amyloid precursor protein transgenic mouse model of Alzheimer’s disease, a recent study found that reducing endogenous ASYN restored the levels of Rab3a and Rab5 proteins^11^. Reduction of endogenous ASYN rescued deficits in neurotrophic factors and prevented the degeneration of cholinergic neurons in this model^11^. Thus, ASYN plays an important role in many aspects of endocytic processes. It is unclear, however, how these processes are affected by excessive accumulation of ASYN that results in neuronal dysfunction under the setting of PD pathogenesis.

ASYN has also been implicated in other proteinopathies such as familial Alzheimer’s disease (AD) and dementia with Lewy bodies (DLB), in which pathogenic tau species (e.g. hyperphosphorylated forms or pTau) are believed to contribute to these conditions. Similar to tau, ASYN has a strong propensity to misfold and recent studies have suggested that ASYN may interact with tau to form deleterious hetero-oligomers for initiating and spreading of neurodegeneration in these diseases^14^. These studies suggest that Tau is an important mediator in transmitting neuronal toxicities of ASYN.

In the present study, we investigated if ASYN induced pTau and endocytic dysfunction in cortical neurons at embryonic stages using the human ASYN transgenic mouse model of PD. We used live imaging of axonal transport of Quantum-dot-labeled brain-derived neurotrophic factor (QD-BDNF) to examine possible mechanism(s) by which accumulated ASYN impacted axonal function in cultured E18 cortical neurons of ASYN-GFP transgenic mouse embryos from Line 78 PD mouse model^15^. Although the level for pTau showed no increase at this stage, we observed that expression of ASYN-GFP induced endocytic dysfunction by upregulating the level of activated Rab5 and Rab7. We also found that ASYN-GFP potentially impaired retrograde transport of BDNF by interacting with the retrograde motor protein dynein, leading to neuronal atrophy. Our study suggests that ASYN-induced axonal dysfunction occurs early in the pathogenesis of PD.

## Materials and Methods

### Animals

All animal studies have been approved by the Institutional Animal Care and Use Committee of University of California San Diego. All experimental procedures were performed in accordance with relevant guidelines and regulations established by NIH Guide for the Care and Use of Laboratory Animals. The PD mouse model used in this study, Line 78, expresses a human-α-synuclein-GFP transgene under the PDGF-β promoter (PDGF-β-ASYN-GFP)^16^. The synuclein knockout (ASYN^−/−^) mice were obtained from Jackson laboratories. All animals were maintained and bred according standard procedures.

### Genotyping

The line 78 pregnant mice carried a mixture of wild type and transgenic embryos. The GFP^+^ E18 embryos were screened using a “GFP flashlight” (Nightsea). Those that were visibly “green” were deemed as transgenic, remaining embryos were designated as non-transgenic. Nontransgenic littermates were used as controls. The SYN^−/−^ pregnant mice produced embryos all with the same SYN^−/−^ genotype.

### Chemicals, reagents, media, antibodies and plasmids

Hanks Balanced Salt Solution, neurobasal media, trypsin, B27, GlutaMax, penicillin-streptomycin, streptavidin-QD655 conjugates were purchased from Invitrogen. FBS was purchased from Phoenix Research Products. HEPES, poly-L-lysine, DNase I, and GTP agarose beads were purchased from Sigma-Aldrich. Protein IgA/G agarose beads were purchased from Amersham Biosciences.

Rabbit anti-Akt, rabbit anti-pAkt and rabbit anti-ERK1/2 were purchased from Cell Signaling Technologies. Rabbit anti-pTrkB (pY490) was kindly provided by Dr. Moses Chao (NYU). Mouse anti-TrkB and mouse anti-α-synuclein were purchased from BD. Mouse anti-pErk1/2, mouse anti-GFP, mouse anti-dynein (DIC-74) and rabbit anti-Rab5B were purchased from Santa Cruz. Mouse anti-Rab7 was purchased from Abmart. Mouse anti-GAPDH and mouse anti-β-actin were from GeneTex. Rabbit anti-Tau(pT181), rabbit anti-Tau(pS199), anti-Tau(pT205), anti-Tau(pT231), anti-Tau(pS396/404) and rabbit anti-Tau were all purchased from Genscript (Piscataway, NJ). Both goat anti-rabbit and anti-mouse IgG–HRP conjugates were purchased from Jackson ImmunoResearch Laboratories Inc.

For transient transfection, a full-length alpha-synuclein cDNA was generated by PCR using a human brain cDNA library and was cloned into the pEGFP-N1 vector between Xho-I and Kpn-I. The plasmid was verified by sequencing^17^. The GFP plasmid from Clontech was used as a control.

### Primary neuron cultures and PC12 cell culture

Cortical neurons were dissected and cultured from either PD transgenic or non-transgenic mouse embryos, or from SYN^−/−^ mouse embryos or from E18 rat embryos following published protocols^15, 18, 19^. Briefly, cortex was dissected, dissociated, resuspended in Neurobasal with 10% FBS, B27, GlutaMAX and plated in the same media overnight. The media were then replaced with maintenance medium (Neurobasal with B27, GlutaMAX) the next day. Two-thirds of the media was replaced every 2–3 days until experiments were concluded.

For live imaging experiments, cortical neurons were cultured into microfluidic chambers with 450 μm microgrooves (Xona microfluidics), and live imaging of axonal transport assays were carried out at DIV7 (Days In Vitro)^15, 18, 20^. For signaling studies, mass cultures of cortical neurons were starved for 2 hrs in neurobasal media and were stimulated with 50 ng/ml BDNF at different time points^18^. Neurons were harvested and lysed in PBS containing 1% NP-40, 0.1% SDS, 0.1% deoxylcholate, 1 mM PMSF (phenylmethylsulfonyl fluoride), 0.2 mM sodium orthovanadate and protease inhibitor cocktail (Roche).

For transient expression studies, rat E18 cortical neurons (DIV4) were transfected with either pGFP or pASYN-GFP expression vectors using LipoFectamine 2000. 72 hrs post transfection, images of cells were captured with a 20x objective lens using a Leica DMi8 Live Imaging Microscope. PC12 cells were maintained, transfected and imaged as described^20–22^. The mCherry-Rab5^WT^ plasmid used in co-transfection experiments in PC12 cells was reported earlier^20^. The soma sizes of untransfected, GFP- and ASYN-GFP expressing neurons and the size of mCherry-Rab5^WT^ endosomes were measured using ImageJ^20^.

### Live imaging of Quantum dots-labeled BDNF (QD-BDNF)

Mouse purified monobiotinylated BDNF (mBtBDNF) was produced as previously published^15, 23^. To prepare the QD-BDNF conjugates, the mixture of 100 nM mBtBDNF and 100 nM streptavidin-QD655 was incubated on ice for 30 minutes. Prior to live imaging, cortical neurons were starved with serum-free neurobasal media for 3 hours. QD-BDNF was added to the axonal compartment at a final concentration of 0.2 nM and incubated for 3 hours. Time-lapse images were captured at 1 frame/sec for a total of 2 minutes per movie using a Leica DMI6000B inverted microscope, which is equipped with an environmental chamber (37 °C, 5% CO_2_). Fluorescent images as well as DIC images were also captured for analysis. Quantitation of axonal transport of BDNF was performed using Image J^15, 18, 20, 24^. Briefly, the direction of movement was determined by the angle measurement for each line on kymographs using cut-offs as specified below: retrograde (−1 to −89.4), anterograde (<−90.6), and stationary (90 ± 0.5). The distance traveled by one particular BDNF signal divided by the total time including pausing time is designated as the average transport velocity, whereas the distance divided by the total time minus pausing time is defined as the moving velocity. Pauses are the percentage of the time of a BDNF signal staying at stationary over the total time. All live-imaging experiments were performed blindly to minimize bias.

### Rab GTP-pull down assay

Published methods were followed to quantitate the level of GTP-bound of Rab5 or Rab 7^18, 20, 25^. Briefly, mouse brain tissues were lysed (50 mM Tris-HCl pH 7.5, 250 mM NaCl, 5 mM Mg Acetate, 0.5% Triton X-100, and protease inhibitor) and incubated on ice for 30 minutes. All lysates were centrifuged to produce supernatants. After determining the concentration (BioRad Protein Assay), an aliquot of supernatants (15–20 μl) was saved as the loading control to measure the level of total Rab proteins. Equal amounts of supernatants were taken out and incubated with GTP-agarose beads overnight at 4 °C with rotation. The beads were washed and boiled. The amount of Rab5-GTP was measured by SDS-PAGE and blotted with anti-Rab5 antibody.

### Immunoprecipitation

Mouse brain tissues were homogenized (20 mM Tris-HCl pH 7.4, 150 mM NaCl, 1% Triton X-100, 1 mM sodium orthovanadate, 0.2 mM PMSF and protease inhibitor cocktail) and incubated on ice for 30 minutes, followed by a centrifugation for 30 minutes at 4 °C. The supernatant was removed and protein concentration was assayed (BioRad Protein Assay). For immunoprecipitation, 10 μl of mouse anti-dynein intermediate chain (DIC-74) was added to the supernatant and immunoprecipitates were isolated by the addition of protein A/G agarose beads^26^. Following overnight incubation, immunoprecipitates were washed three times with lysis buffer and associated proteins were detected by SDS-PAGE/immunoblotting with a mouse anti-GFP antibody or a mouse anti-α-synuclein antibody.

### SDS-PAGE immunoblotting

Equal amounts of proteins were separated on SDS-PAGE gels and then electrotransferred to PVDF membranes. The membranes were blocked with 5% nonfat milk and probed with specific antibodies following established protocols^18, 20, 25, 26^. All blots were captured using ChemiDoc XRS + (Bio-Rad), and only blots within linear ranges were quantitated using ImageLab 3.0.1 software (Bio-Rad)^18, 20^. All original blots, when possible, full size uncut blots, are presented in Supplemental Information.

### Statistics

All experiments were repeated at least 3 times independently. Data represent mean ± SEM. Statistical analyses and calculation of P values were performed using Prism5 (GraphPad Software); Student’s t test was used for pairwise comparisons. One-Way ANOVA with Bonferroni’s post test was used for frequency distribution analysis. P values less than 0.05 were considered statistically significant, and P values less than 0.01 were considered statistically highly significant.

## Results

### Significant deficits in retrograde axonal transport of BDNF in Line 78 E18 cortical neurons

Strong evidence suggests that increased accumulation of ASYN affects intracellular vesicular trafficking^10, 11^. We hypothesized that axonal trafficking and function was one of the neuronal processes most susceptible to ASYN toxicity. To examine the possible impact of increased α-synuclein on axonal transport, we used E18 primary cortical neurons from Line 78 transgenic mice (PDGF-β-ASYN-GFP), in which the expression of ASYN-GFP was driven by the PDGF-β promoter^16, 27^. E18 primary cortical neurons from transgenic mice and non-transgenic littermate controls were cultured in microfluidic chambers, by which axons are separated from the corresponding cell bodies^15, 18^. To examine axonal function, we carried out live imaging experiments to measure retrograde axonal transport of quantum-dot labeled BDNF (QD-BDNF), an assay well established in our laboratory^15, 20, 23, 28, 29^.

**Figure 1:**
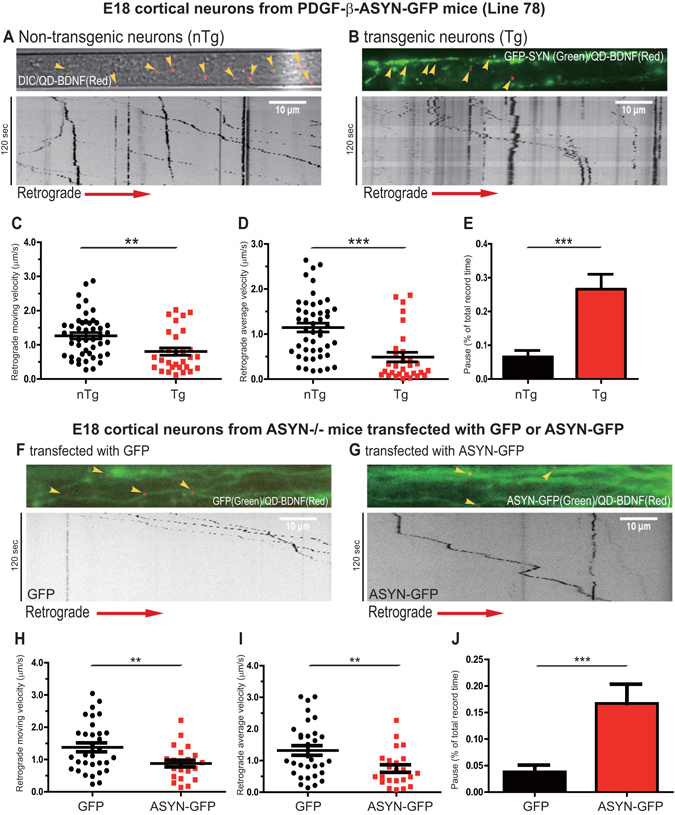
Expression of GFP-ASYN induced retrograde axonal transport deficits of BDNF in E18 cortical neurons. E18 cortical neurons from Line 78 (PDGF-β-ASYN-GFP) mouse embryos were dissected and cultured in microfluidic chambers as described in Materials and Methods. At DIV7, neurons were deprived of BDNF for 3–4 hrs, QD-BDNF (0.2 nM final concentration) was added to the axonal chambers. Axonal transport of QD-BDNF was captured by live imaging. Kymographs were generated from time-lapsed image series. Representative image of QD-BDNF within axons of non-transgenic (**A**) and transgenic neurons (**B**) are shown. Axonal transport parameters: the retrograde moving velocity (**C**), average velocity (**D**) and pausing time (**E**) are quantitated and presented. Data were obtained from 10 non-transgenic neurons and 20 GFP-positive transgenic neurons. In **F–J**, E18 cortical neurons from ASYN knockout (−/−) mice were dissected and cultured. At DIV4, cultures were transfected with either GFP or ASYN-GFP and BDNF transport assays were carried out as described above. Representative images of transfected axons and kymographs are shown: **F** for GFP; **G** for ASYN-GFP. The retrograde moving velocity (**H**), average velocity (**I**) and pausing time (**J**) were quantitated and presented. Data were obtained from 15 GFP-expressing neurons and 15 GFP-ASYN-expressing neurons. All data are analyzed using Prism Graphpad 6.0. The p values were obtained using student t-test. *p* < 0.05 (**); *p* < 0.01 (***); n.s. = non-significant.

Strong expression of the ASYN-GFP transgene was observed in ~10% of cortical neurons at this stage, as reported previously^16, 27^. Strong GFP signals thus indicated robust expression of ASYN and vice versa. We therefore focused our analysis on GFP-positive neurons/axons in cultures from Line 78 transgenic samples and compared them to GFP-negative neurons/axons in the same culture to gauge the impact of increased levels of ASYN on axonal transport (Fig. 1B versus A). Kymographs of live imaging series of axonally transported QD-BDNF were generated; representative images with corresponding kymographs are shown (Fig. 1A–E).

Analyses of kymographs of axonally transported QD-BDNF signals revealed significant differences between GFP-negative and GFP-positive axons. In GFP-negative axons, the movement of QD-BDNF was persistent and processive (Fig. 1A), a pattern fully consistent with earlier findings^18, 30^. However, QD-BDNF puncta in neurons derived from GFP-positive axons showed much more frequent pauses and short switches in moving directions, in addition to processive movements (Fig. 1B). Measurements of moving (i.e. instantaneous) and average velocities were performed and the results were plotted respectively. When compared with GFP-negative neurons, there were remarkable decreases in both the rate of transport (Fig. 1C) and average (Fig. 1D) velocities of retrograde transport of QD-BDNF in axons of GFP-positive neurons. The transport velocities of QD-BDNF puncta in GFP-positive neurons were significantly reduced by approximately 36.5% (*p* = 0.002) relative to GFP-negative neurons. The average speeds detected in GFP-positive neurons were also reduced to 42.7% (*p* < 0.0001) compared to those in GFP-negative neurons. We further examined and computed the pause events. For each QD-BDNF signal, we counted all the pause events, calculated the total pause time, and then obtained the percentage of total pause time in the total recording period (120 seconds). As shown in Fig. 1E, the pause periods were significantly increased for QD-BDNF signals (26.6%, *p* < 0.0001) within axons of GFP-positive neurons in comparison with GFP-negative neurons.

### BDNF retrograde transport deficits correlate with the expression of ASYN in ASYN^−/−^ E18 cortical neurons

To further establish the link between axonal transport deficits of BDNF with the expression of ASYN, we next prepared primary cortical neuronal cultures from ASYN^−/−^ mice and transfected them with either a GFP plasmid vector or a plasmid encoding GFP-tagged wildtype human ASYN on day *in vitro* 4 (DIV4). QD-BDNF retrograde transport assays were performed at DIV7 (Fig. 1F–J). QD-BDNF puncta in ASYN^−/−^ neurons expressing a GFP-tagged wildtype human α-synuclein (ASYN-GFP) construct stopped more frequently and demonstrated considerable switches of directions during the moving period than those in ASYN^−/−^ neurons transfected with the GFP plasmid vector (Fig. 1F and G). Further analyses revealed that both the moving and average velocities of retrograde transported BDNF in ASYN^−/−^ neurons expressing ASYN-GFP were significantly reduced to 63.6% (p = 0.0088) and 56.7% (p = 0.0077) (Fig. 1H and I). The reduced velocities were accompanied with a marked, concomitant increase of pause period (p = 0.0004), when compared with neurons transfected with the GFP control vector (Fig. 1J). Taken together, these observations have demonstrated a role of increased levels of ASYN in disrupting retrograde axonal transport deficits of BDNF.

### The level of pTau species is not increased in Line 78 E18 cortical neurons

Microtubule associated protein tau is expressed predominantly in the axons of neurons where it promotes assembly and stabilization of microtubules^31^. In pathological conditions, increased pTau may cause microtubule destabilization and impair axonal transport^32^. Accumulated/aggregated α-synuclein has been shown to induce pTau and decrease microtubule stability^33^.

**Figure 2:**
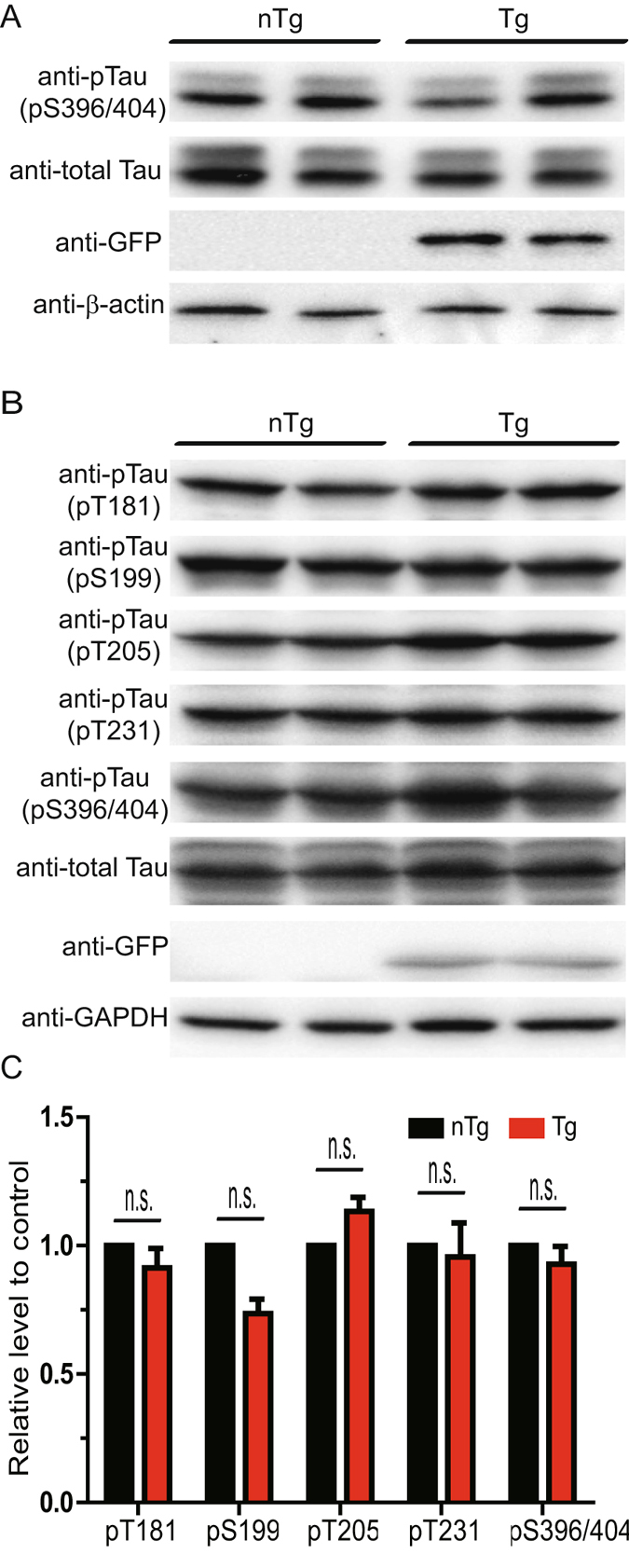
No increase pTau species was detected in Line 78 E18 neurons and embryos. E18 cortical neurons from mouse cortical neurons from Line 78 transgenic embryos and nontransgenic littermates were cultured in 12 well plates. Neurons were collected at DIV4 and protein lysates were generated and analyzed by SDS-PAGE/immunoblotting with antibodies indicated (**A**). In **B**, brain homogenates from 8 month-old Line 78 transgenic embryos or non-transgenic littermates were analyzed using indicated specific antibodies as in **A**. In **C**, the protein levels of pTau were quantitated and expressed as percentage of change relative to changes observed in control mice. No significant changes were seen between transgenic and non-transgenic samples. At least three independent experiments were performed for all the studies presented. All original, full size, uncut blots are presented in Supplemental Information.

To determine if pTau could account for ASYN-induced axonal transport deficits in E18 cortical neurons from Line 78 mice, we examined the levels of pTau by SDS-PAGE/immunoblotting with specific antibodies against pTau at different sites^34^. We first tested E18 cortical neurons cultured from either Line 78 PD transgenic mice or non-transgenic controls at DIV7, at which time retrograde axonal transport of BDNF was significantly impaired in cortical neurons of Line 78 (Fig. 1). To our surprise, we did not observe a significant increase in the level of Tau (pS394/404) in E18 cortical neurons cultured from Line 78 PD transgenic mice in comparison to non-transgenic controls (Fig. 2A). To further confirm if that was not an artifact associated with *in vitro* E18 cortical neuronal culture, we generated and analyzed brain lysates from 8 month-old Line 78 transgenic mice and their non-transgenic littermates. Our immunoblotting results showed no significant changes in the level of pTau as detected with a panel of site-specific pTau antibodies (Fig. 2B,C). These results suggest that pTau unlikely contributes to axonal transport defects in E18 cortical neurons from the Line 78 PD mouse model.

### Increased level of ASYN enhanced Rab5 activity

Recent evidence has revealed that members of the Rab GTPase family such as Rab5 play a key role in regulating axonal trafficking^20, 35–37^. We next decided to examine if abnormal activation of Rab5 could account for the axonal transport deficits in Line 78 cortical neurons. There are two conformational states of Rab5 cycling: the GTP-bound active form and the GDP-bound inactive form. In the active form, multiple effectors are recruited and involved in the dictation of endocytosis, trafficking and sorting of surface cargos. Residing in early endosomes, Rab5 is proposed to be a crucial mediator in the transport of signaling endosomes of neurotrophic factors^38^. Increased level of GTP-bound Rab5 has been shown to impede retrograde axonal transport of NGF^20^ and BDNF^18^.

**Figure 3:**
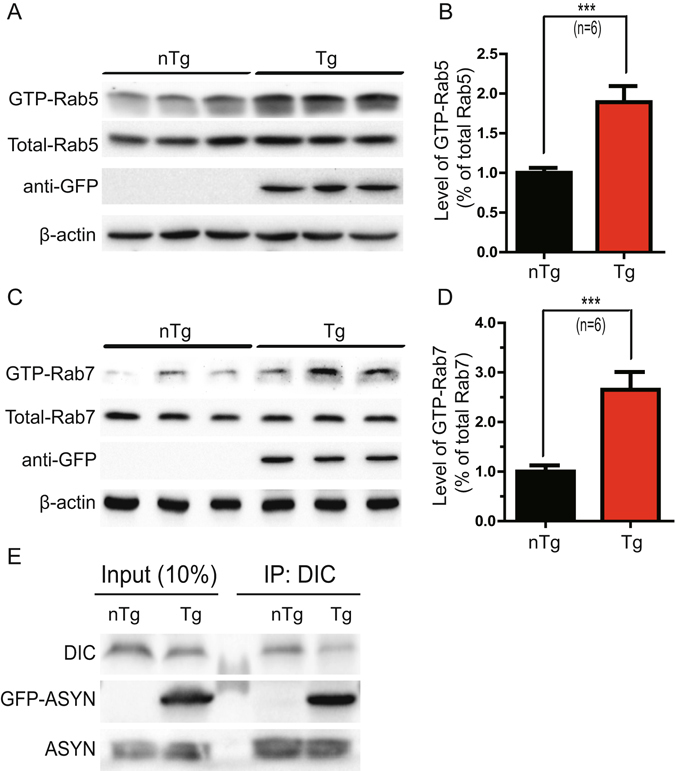
Increased level of GTP-bound Rab5 and Rab7 was detected in Line 78 E18 brain samples. Brain lysates were incubated with GTP-agarose beads to pull down activated GTP-bound Rab5 (**A**) as described in Materials and Methods. The levels of GTP-Rab5 in these samples were quantitated and normalized against internal control. Samples from Line 78 mice show an 89% increase over non transgenic controls (**B**). Similarly, activated Rab7 was pulled down (**C**) and a significant increase of GTP-Rab7 is seen in PDGF-β-ASYN-GFP mice as compared to non-transgenic controls (**D**). For B and D, results are from 6 animals per group. ***p < 0.01. Mouse brain homogenates were immunoprecipitated with an antibody recognizing dynein intermediate chain (DIC). Immunoprecipitates were analyzed by SDS-PAGE/immunoblotting with antibodies as indicated (**E**). At least three independent experiments were performed for all the studies presented. When possible, all original, full size, uncut blots are presented in Supplemental Information. Otherwise, the original blots are presented in Supplemental Information.

**Figure 4:**
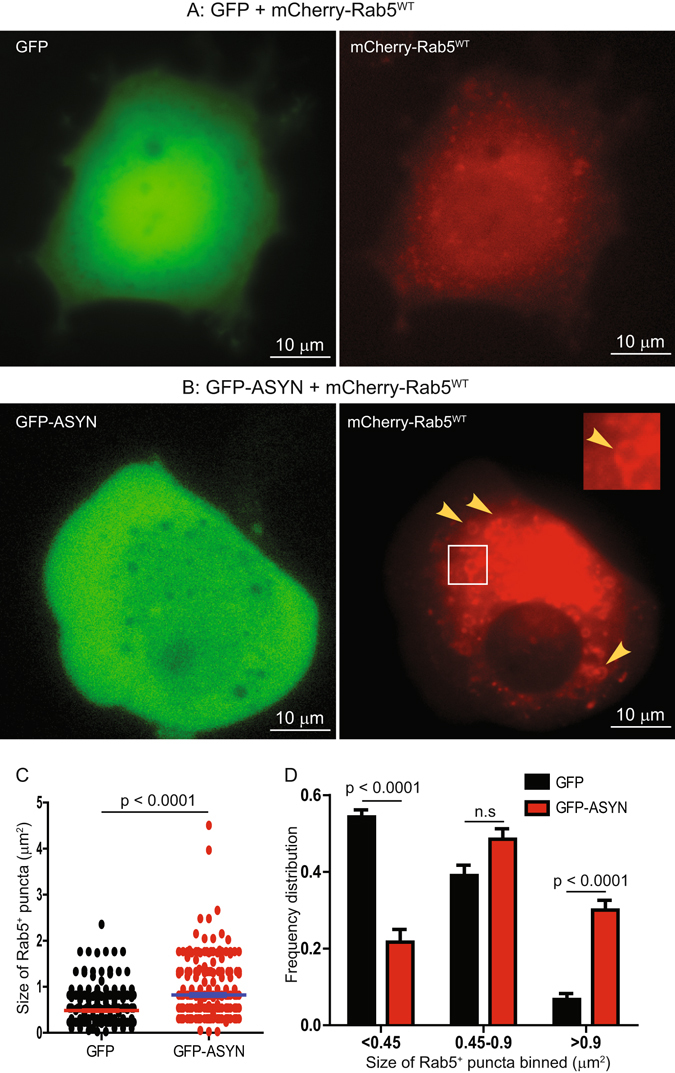
Expression of GFP-ASYN induced enlargement of Rab5 early endosomes in PC12 cells. PC12 cells were transfected with either GFP + mCherry-Rab5^WT^ (**A**) or GFP-ASYN + mCherry-Rab5^WT^ (**B**) for 48 hrs. Enlarged mCherry-Rab5 early endosomes typically seen in GFP-ASYN cells were indicated with arrow heads (**B**). The size of mCherry-Rab5 in **A** and **B** was quantitated and presented in **C;** and the size distribution of the mCherry-Rab5 early endosomes was presented in **D**. The *p* values are indicated and n.s. = non-significant.

To examine whether the activation level of Rab5 was affected by increased level of ASYN, the E18 brains from Line 78 transgenic mice and their non-transgenic littermate controls were collected and lysed. The cleared lysates were incubated with GTP-agarose beads to pull down the GTP-bound form of Rab5, which could be detected by immunoblotting, as described previously^18, 20, 25, 39^. Our results showed a significant increase in the level of GTP-bound Rab5 in the transgenic mice brains (*p* = 0.0072) (Fig. 3A and B). An increase in the level of GTP-bound Rab5 is often accompanied by enhanced homotypical fusion of Rab5 positive early endosomes^20, 28^. To determine if increased ASYN also induced enlarged Rab5 early endosomes, we co-transfected GFP or GFP-ASYN with mCherry-Rab5^WT^ into PC12 cells. The signal for mCherry-Rab5^WT^ increases in response to activation of Rab5, as we have previously demonstrated that increased levels of GTP-bound Rab5 led to enlargement of mCherry-Rab5^WT^-positive puncta^20^. When co-transfected with GFP-ASYN, the size of mCherry-Rab5^WT^-positive endosomes became markedly enlarged in comparison with co-transfection with GFP alone (Fig. 4B versus A). Quantitative analysis confirmed that the average size of mCherry-Rab5^WT^-positive endosomes was 0.823 ± 0.029 μm^2^ for GFP-ASYN expressing cells, that was significantly greater than 0.485 ± 0.014 μm^2^ for GFP control cells (Fig. 4C) (p < 0.0001). A detailed size distribution analysis revealed that expression of GFP-ASYN, in comparison to GFP alone, induced a marked increase in large size (>0.9 μm^2^, *p* < 0.0001) with a concomitant decrease in smaller size (<0.45 μm^2^, *p* < 0.0001) of mCherry-Rab5^WT^ positive endosomes (Fig. 4D). The population of mCherry-Rab5^WT^ positive endosomes with median size (0.45–0.9 μm^2^) did not show an appreciable difference between the two groups (Fig. 4D). We thus conclude that increased level of ASYN increases the level of activated Rab5 and induces enlargement of Rab5-positive early endosomes.

Interestingly, Rab7, another small GTPase that regulates endocytic trafficking of neurotrophic factor signals^25^ was also increased in the GTP-bound state in the brain lysates from transgenic mice (*p* = 0.0072) (Fig. 3C and D). These results suggest that increased level of ASYN enhanced the activation level of Rab5 and Rab7. Consistent with previous findings, our study thus suggests an important role of ASYN in disrupting endocytic trafficking of BDNF.

### GFP-ASYN interacts with dynein

Cytoplasmic dynein serves as the motor that drives the retrograde transport of signaling endosomes along microtubule tracks^26^. It is possible that excessive ASYN could interact with dynein to impact retrograde axonal transport of BDNF (Fig. 1). To investigate this possibility, we performed immunoprecipitation experiments using mouse brain lysates from Line 78 mice and their littermate non-transgenic controls using an antibody that specifically recognizes the dynein intermediate chain (DIC) to pull down the DIC-containing protein complex. The immunoprecipitates were analyzed by immunoblot using antibodies to detect either overexpressed ASYN-GFP or endogenous ASYN, respectively. Both overexpressed and endogenous ASYN co-immunoprecipitated with dynein intermediate chain (Fig. 3E), consistent with previous reports^40^. Our findings suggest that excessive accumulation of ASYN could potentially sequester dynein motor proteins to affect retrograde axonal transport.

### Increased level of ASYN inhibit TrkB-mediated signaling

**Figure 5:**
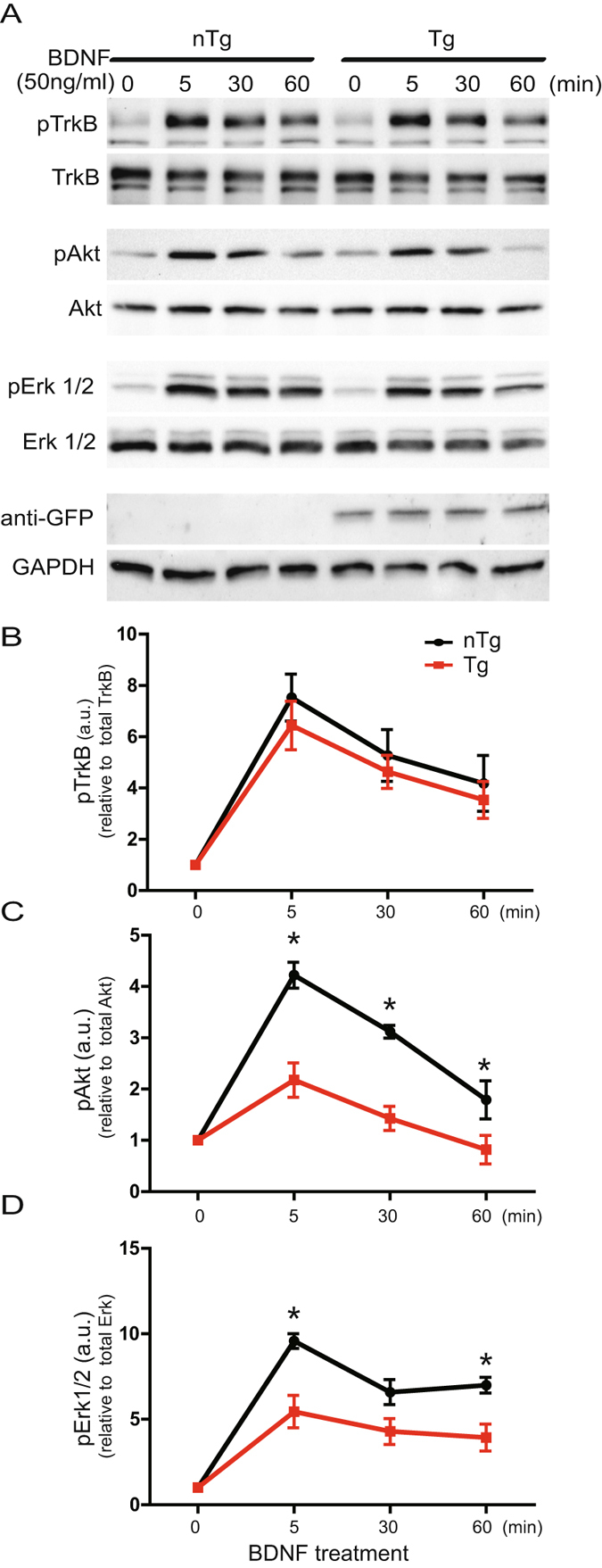
Increased level of ASYN inhibited BDNF/TrkB-mediated down-stream signaling. Mouse E18 cortical neurons were cultured in 12-well plate (mass cultures) and stimulated with 50 ng/ml at DIV4. Equal amounts of protein lysates were analyzed by SDS/PAGE and immunoblotting with specific antibodies as indicated (**A**). Semi-quantitative results are shown for the level of activated TrkB (**B**), activated Akt (**C**) and activated Erk1/2 (**D**). At least three independent experiments were performed for all the studies presented. For these blots, we do not have all original full size, uncut blots. Nevertheless, the original blots are presented in Supplemental Information.

Given that increased levels of ASYN induce deficits of BDNF retrograde transport, we asked whether BDNF downstream signaling was also affected. To test this possibility, we examined BDNF signaling in mass cultures of E18 cortical neurons derived from Line 78 transgenic mice and their littermate non-transgenic controls. Primary cultured cortical neurons at DIV7 were stimulated with BDNF (50 ng/ml) for different time points, then collected and examined by immunoblotting for the activation of the TrkB receptor as well as two downstream signaling pathways, Akt (part of the Akt/PI3K signaling pathway) and Erk1/2 (part of the MAPKK signaling pathway). Following addition of BDNF (50ng/ml), the activation of TrkB peaked at 5 minutes followed by a progressive attenuation of response over 60 minutes, which were observed in both non-transgenic and transgenic neurons. There was no significant difference in the level of pTrkB between the two groups (Fig. 5A and B). Both pAkt and pErk1/2 levels were significantly lower in transgenic neurons at all time points tested for pAkt and at 5 and 60 min for pErk1/2 (Fig. 5A, C and D). Our results suggest that elevated α-synuclein not only impairs retrograde axonal transport of BDNF but also suppresses BDNF-mediated trophic signaling cascades.

### Increased level of ASYN induces cortical neuronal atrophy

**Figure 6:**
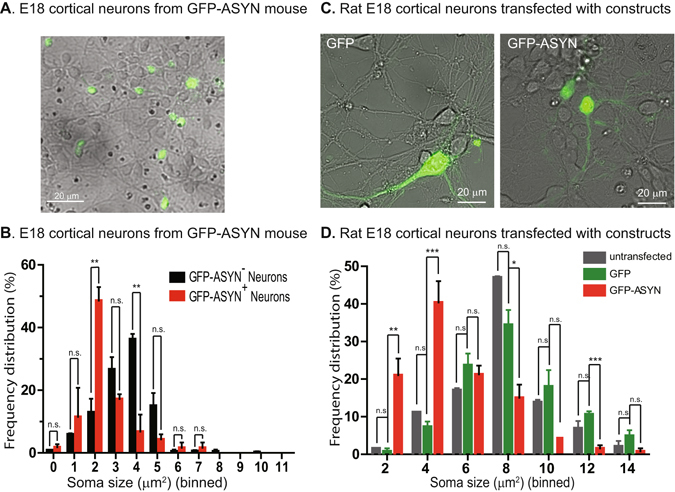
Increased level of ASYN induced cortical neuronal atrophy. E18 cortical neurons from either Line 78 (**A**,**B**) were dissected and cultured as in Fig. 1. At DIV7, the cultures were analyzed by microscopy. Both GFP and phase contrast images were captured and merged. As shown in **A**, not all neurons expressed ASYN-GFP. The soma sizes of GFP-positive neurons (n = 40) and GFP-negative neurons (n = 112) were measured in ImageJ. Histogram analysis of the size distribution of GFP-positive versus GFP-negative neurons is shown in **B**. In **C**,**D**, rat E18 cortical neurons were dissected and cultured as described in Materials and methods. Neurons were transfected with either GFP or ASYN-GFP using LipoFectamine2000. The cultures were analyzed by microscopy 72 hrs post transfection. Both GFP and phase contrast images were captured and merged (**C**). The soma sizes of untransfected neurons (n = 188), GFP-positive neurons (n = 122) and ASYN-GFP neurons (n = 127) were measured in ImageJ. Histogram analysis of the size distribution of untransfected, GFP-positive and ASYN-GFP neurons is shown in **D**. The differences between ASYN-GFP and untransfected neurons and between ASYN-GFP and GFP neurons are highly significant (***), while the difference between untransfected and GFP neurons is not significant (One-Way ANOVA with Bonferroni’s post-test).

Retrograde axonal signaling and trafficking of trophic factors such as NGF and BDNF has been shown to play an important role in maintaining the trophic status of their responsive neurons^20, 23^. Based on our findings that retrograde axonal transport of BDNF was reduced and BDNF-induced Akt- and Erk1/2-signaling was inhibited in E18 cortical neurons of the PDGF-β-ASYN-GFP mice (Line 78), we predicted that overexpression of ASYN would reduce the soma size of these neurons. We measured the size of neurons expressing ASYN-GFP and compared them to sizes of neurons that were not expressing ASYN-GFP; histogram analysis of size distribution revealed a reduction in neuronal soma size in neurons expressing ASYN-GFP (GFP-ASYN^+^) versus GFP-ASYN^-^ cells (Fig. 6A and B).

To confirm if cortical neuronal atrophy was the direct result of overexpression of ASYN, we cultured primary cortical neurons from E18 rat embryos transfected with either GFP or ASYN-GFP at DIV4. The soma sizes of untransfected, GFP-transfected, and ASYN-GFP-transfected neurons were measured 72 hrs post transfection. Histogram analysis of size distribution revealed that the size of ASYN-GFP expressing neurons was significantly smaller than the size of either GFP-expressing neurons or untransfected neurons (Fig. 6C,D), though there was no significant difference between GFP-expressing neurons and untransfected neurons. Based on these results, we conclude that while GFP expression does not affect the soma size of cortical neurons, expression of ASYN-GFP induces significant atrophy in cortical neurons.

## Discussion

Increasing evidence suggests that defective axonal transport is attributed to early pathogenesis of PD; however, the underlying mechanism remains to be determined. Here, we have demonstrated that increased level of ASYN impeded retrograde axonal transport of BDNF and inhibited BDNF-induced trophic signaling at the embryonic stage. We further showed that increased expression of ASYN interacts with dynein motor and induces endosomal dysfunction by enhancing the level of activated Rab proteins (Rab5, Rab7), which may contribute to transport impairment. Surprisingly, we did not detect abnormal phosphorylation of tau, given that tau has been implicated in contributing importantly to synucleinopathies^14, 31–34, 41, 42^. We speculate that axonal dysfunction at embryonic stage in neurons of the PD mouse models examined herein results from cellular events independent of tau. Our study has revealed a novel mechanism by which excessive accumulation of ASYN induces axonal transport deficits and causes neuronal atrophy in the very early stages of PD.

BDNF, a member of the neurotrophic factor family, supports the differentiation, maturation and survival of neurons^43–45^, and has the potential to effectively treat PD^46–49^. We focused our effort on retrograde axonal transport and signaling of BDNF. Using a novel *in vitro* microfluidic chamber neuronal culture system in combination with a technique for labeling BDNF with Quantum dots, we were able to track and quantitate axonal movements of a single BDNF dimer by live imaging indicating our setup provides a sensitive measurement of axonal function^15, 18, 24^. Alterations and changes in axonal transport behaviors of BDNF, such as transport velocities, average speeds of movement and pauses of BDNF molecules, are indicative of axonal dysfunctions.

We have described altered movement of BDNF in E18 cortical neurons of Line 78 mouse models of PD (Fig. 1A–E). Also transient expression of ASYN in E18 cortical neurons from an ASYN knockout (ASYN^−/−^) mouse model recapitulated the defect in retrograde axonal transport of BDNF (Fig. 1F–J). Therefore, consistent with previous reports^10, 11, 50, 51^, our findings strongly suggest that axonal dysfunction is an early manifestation of ASYN toxicity in PD.

BDNF binds to and activates surface TrkB receptor at the axonal terminus. Following endocytosis, the BDNF/TrkB signaling complex is retrograde transported along the axon to the cell body. The trophic signaling cascades mediated by BDNF/TrkB include the extracellular signal-regulated kinase 1/2 (ERK1/2), phosphatidylinositol 3-kinase (PI3K)/Akt pathways^52^. Axonal delivery of the BDNF/TrkB trophic signaling to the soma is thus crucial for neuronal survival^38^. For instance, the Erk1/2 and PI3K/Akt pathways play a prominent role in neuronal self-repair during very early stages of PD and they enhanced phosphorylation of BAD at Ser136 or Ser112, promoting the release of Bcl-2 and Bcl-xL leading to inhibition of apoptotic events^53^. Our studies have demonstrated that although no significant difference in the phosphorylated level of TrkB by BDNF is seen in transgenic neurons as compared to control groups, the overall activation of Akt and Erk1/2 signals was significantly decreased in transgenic neurons. These findings are consistent with the notion that ASYN disrupts intracellular vesicular trafficking in PD neurons.

Small GTPases such as Rab5 and Rab7 play a critical role in mediating axonal trafficking of neurotrophic factors^12, 15, 18, 20, 22, 25, 44, 54–58^. Elevated Rab5 activity, along with abnormally enlarged early endosomes, was found in Alzheimer’s disease and Down syndrome, which could result in deficient trophic delivery via axonal transport^20, 39^. In the present study, we have observed hyperactivated Rab5 in a PD mouse model overexpressing wildtype ASYN. We thus speculate that increased activation of Rab5 induced by excessive ASYN could account for impaired retrograde transport of BDNF signaling. In addition, we have observed an interaction between ASYN and dynein intermediate chain as reported earlier^40, 59^. Increased ASYN in PD neurons likely sequesters dynein leading to worsening dysfunction of axonal transport, as indicated by a reduction in the transport velocity of QD-BDNF signals within axons. Therefore, ASYN could impact axonal function through increasing the level of activated Rab proteins and sequestering dynein motor.

Strong evidence has pointed to the dysregulation of tau in PD^32, 34, 41, 42, 60^. Hyper-phosphorylated tau, when induced by stress hormones, has been shown to impair retrograde axonal transport of BDNF^24^. In PD and PD related dementia (PDD) cases, hyperphosphorylated tau was found to be co-localized with ASYN in the same aggregates^60^. Previous studies have suggested that ASYN could initiate the early stages of tau fibriillization^42^. A progressive increase in phosphorylated tau levels has been reported in Line 78 mice at 11 months of age^34^, suggesting a potential role of phosphorylated tau in mediating ASYN toxicity. We did not detect marked change in the levels of phosphorylated tau at the embryonic age in the same mouse PD model; however, significant impairment in axonal transport was already seen at the embryonic stage in the mouse model. Therefore, dysregulation of tau may not play a prominent role in leading to defective axonal transport of BDNF in early stages of PD. Our current studies cannot rule out a role for pathogenic tau species in contributing to PD pathogenesis at a later developmental stage.

Dysfunction and degeneration of axons is often an early sign in neurodegenerative diseases including Alzheimer’s disease (AD), Huntington’s disease (HD) and amyotrophic lateral sclerosis (ALS)^19, 20, 51, 57, 58, 61^. Our current study has demonstrated that excessive ASYN induces endosomal dysfunction and impedes retrograde axonal transport is a very early event in the pathogenesis of PD. In addition, exogenously preformed ASYN fibrils were shown to impair retrograde transport^62^. Our findings are consistent with other reports that axonal degeneration has also been observed in the early stages of PD^51, 63, 64^. For instance, notable decreases in the number and velocities of mobile vesicles were seen in primary midbrain neurons overexpressing wildtype ASYN^50^. Further studies to explore the link between ASYN and dysregulated endocytic functions may yield important clues towards the discovery of novel treatment strategy for PD.

## Electronic supplementary material

Supplemental Information
