# Supplementary material for: Synuclein impairs trafficking and signaling of BDNF in a mouse model of Parkinson’s disease
Source: Sci Rep. 2017 Jun 20;7:3868. doi: 10.1038/s41598-017-04232-4 (PMC5478665; doi:10.1038/s41598-017-04232-4)

Synuclein impairs trafficking and signaling of BDNF in a mouse model of Parkinson's disease

Fang Fang[1,2], Wanlin Yang[1,2], Jazmin B. Florio[2], Edward Rockenstein[2], Brian Spencer[2], Xavier M. Orain[2], Stephanie X. Dong[2], Huayan Li[2], Xuqiao Chen[2], Kijung Sung[2], Robert A. Rissman[2,3], Eliezer Masliah[2], Jianqing Ding[1], Chengbiao Wu[2]

[1]Institute of Neurology and Department of Neurology, Ruijin Hospital, Shanghai Jiao Tong University School of Medicine, Shanghai, China

[2]Department of Neurosciences, University of California San Diego, La Jolla, California, USA

[3]VA San Diego Healthcare System, San Diego, CA

Corresponding authors

Jianqing Ding, PhD
Department of Neurology & Institute of Neurology,
Ruijin Hospital,
Shanghai Jiaotong University School of Medicine,
197 Ruijin Er Rd., Building #11, Room #1205,
Shanghai, China 200025
Email: jqding18@163.com
Tel: 011.86.213.418.6594

Chengbiao Wu, PhD
Department of Neurosciences
University of California San Diego
Medical Teaching Facility, Room 312 MC-0624
9500 Gilman Drive, La Jolla, CA 92093-0624
Email: chw049@ucsd.edu
Tel: 1-858-534-0996

Supplemental Information

All original immunoblots that correspond to those in the text are marked and presented below. When possible, full size uncut blots are shown. We also include multiple exposures in the Supplementary Information file for Figure 3E.

FIG 2A

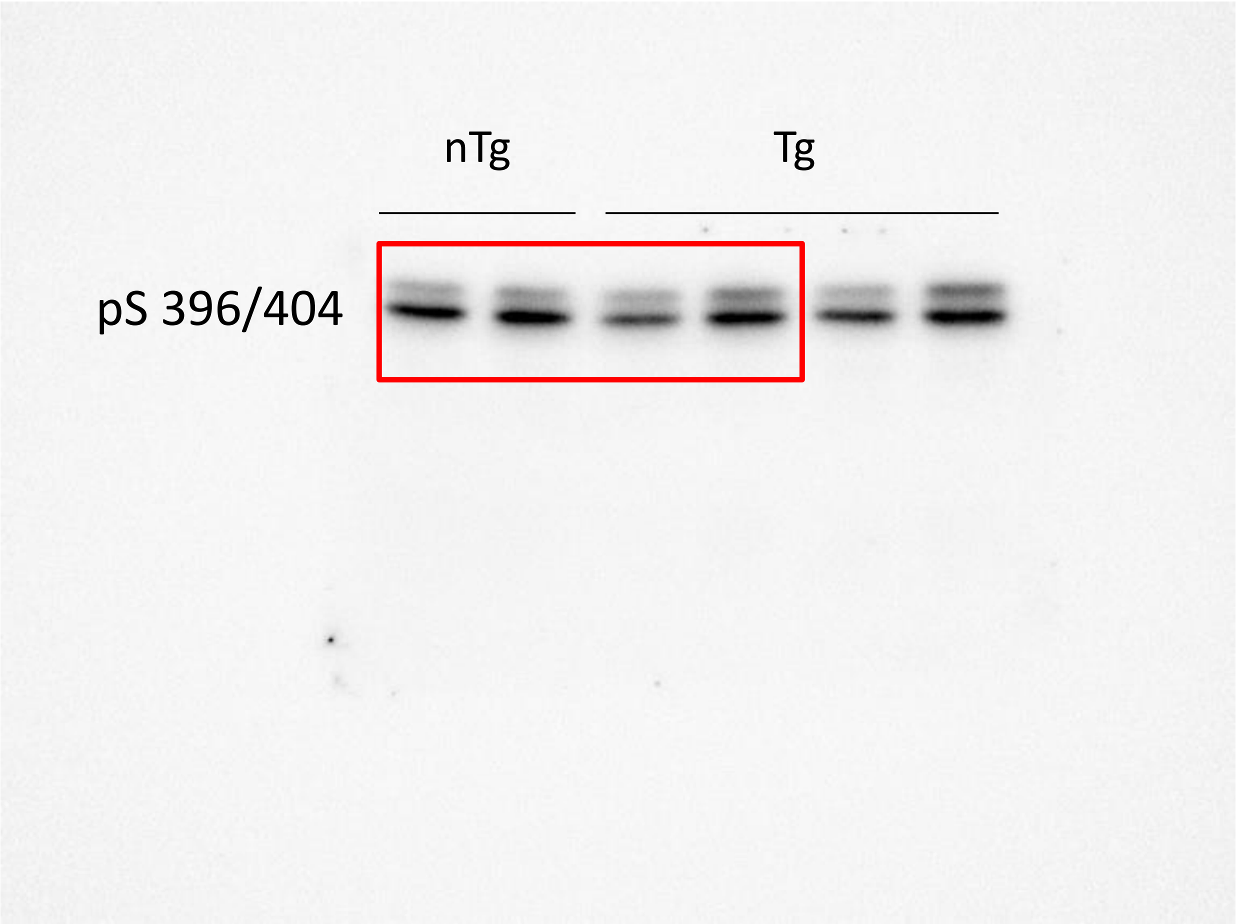

FIG 2A

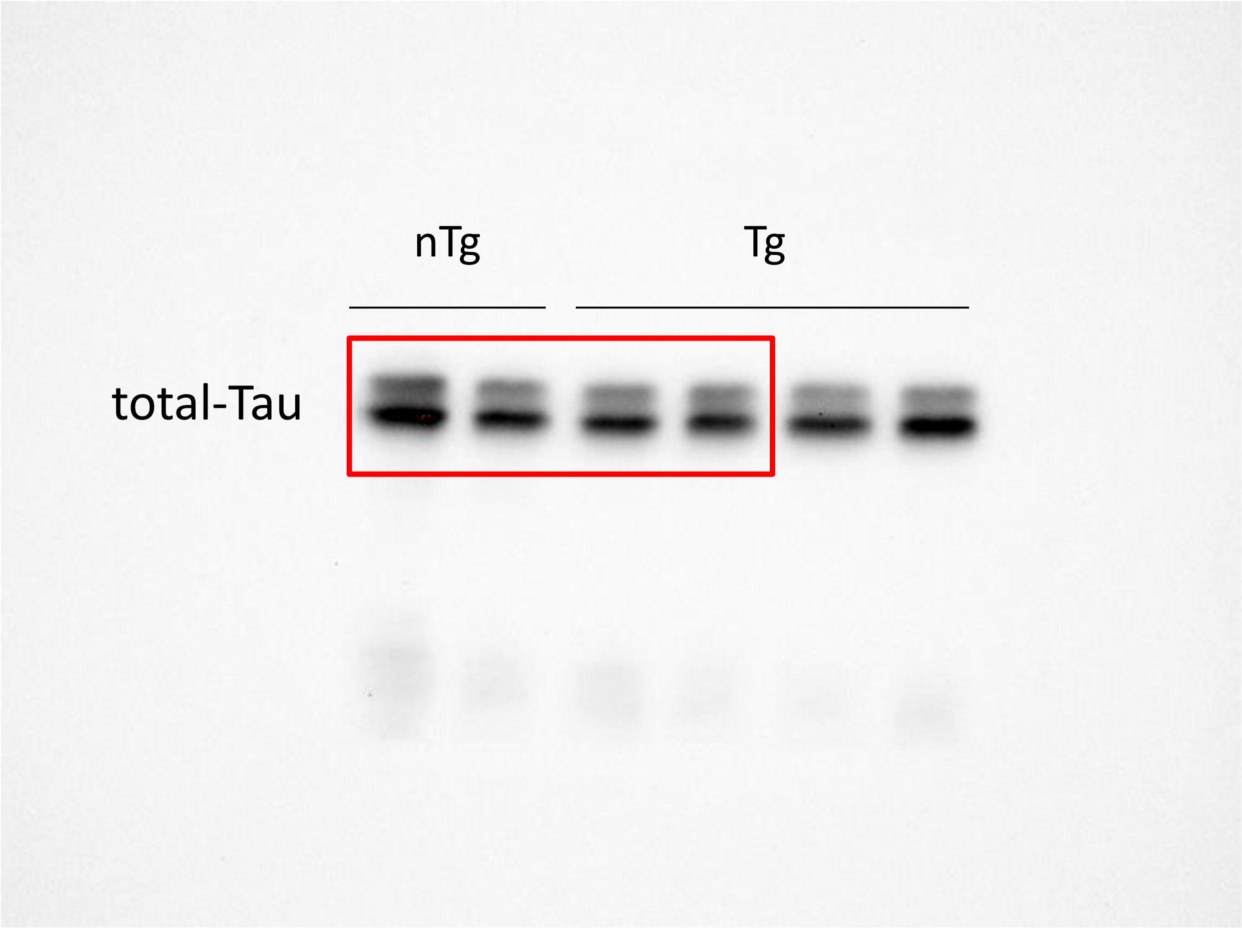

FIG 2A

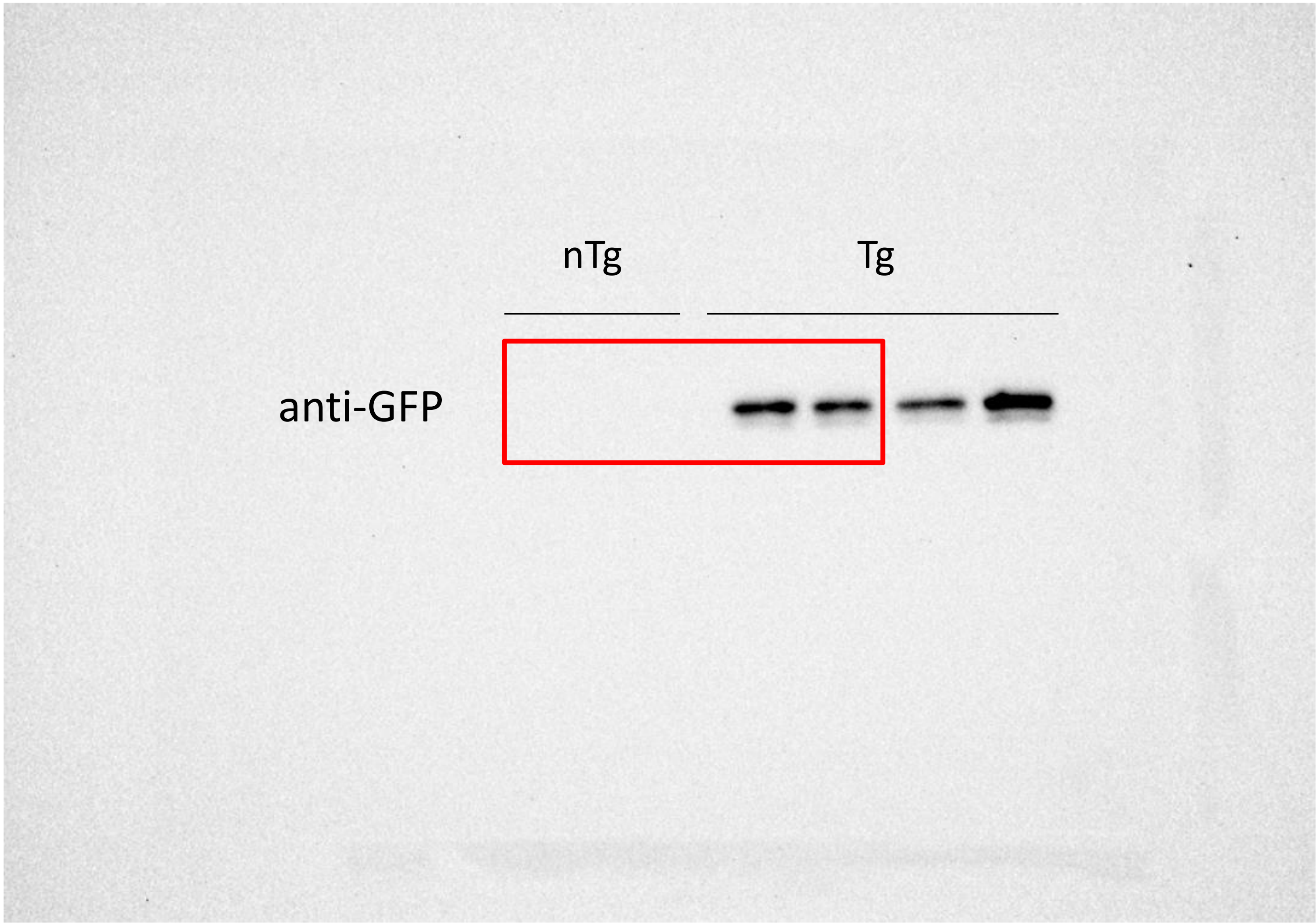

FIG 2A

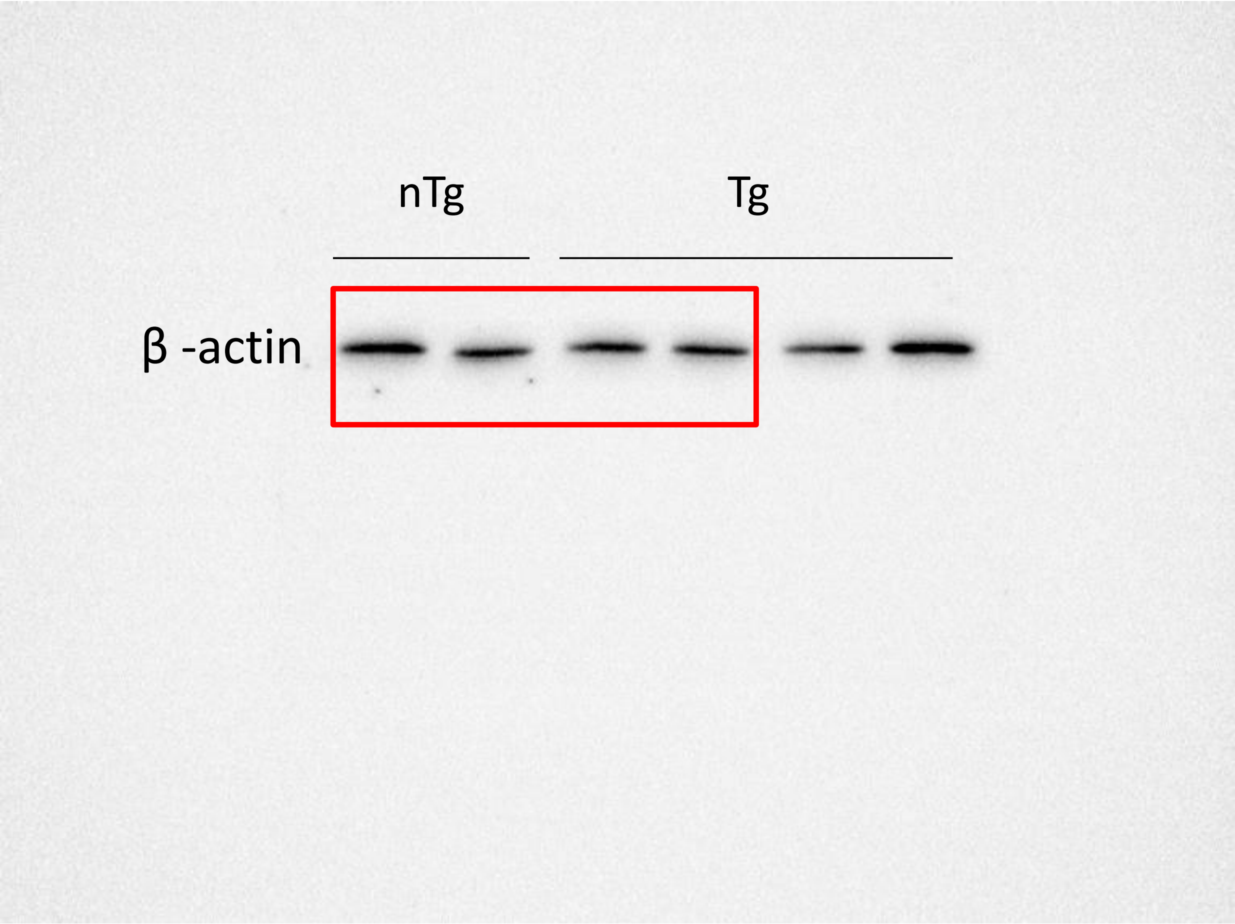

FIG 2B

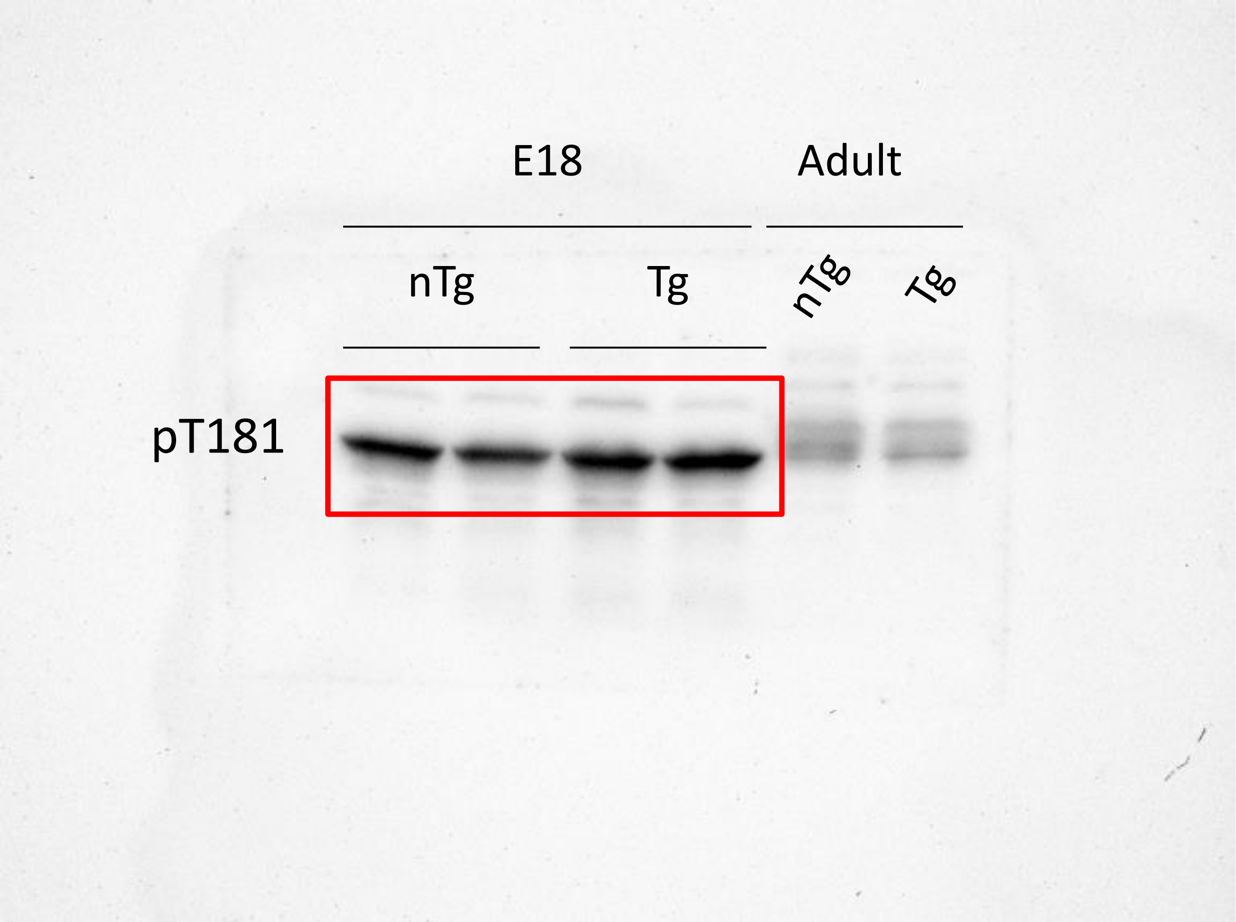

FIG 2B

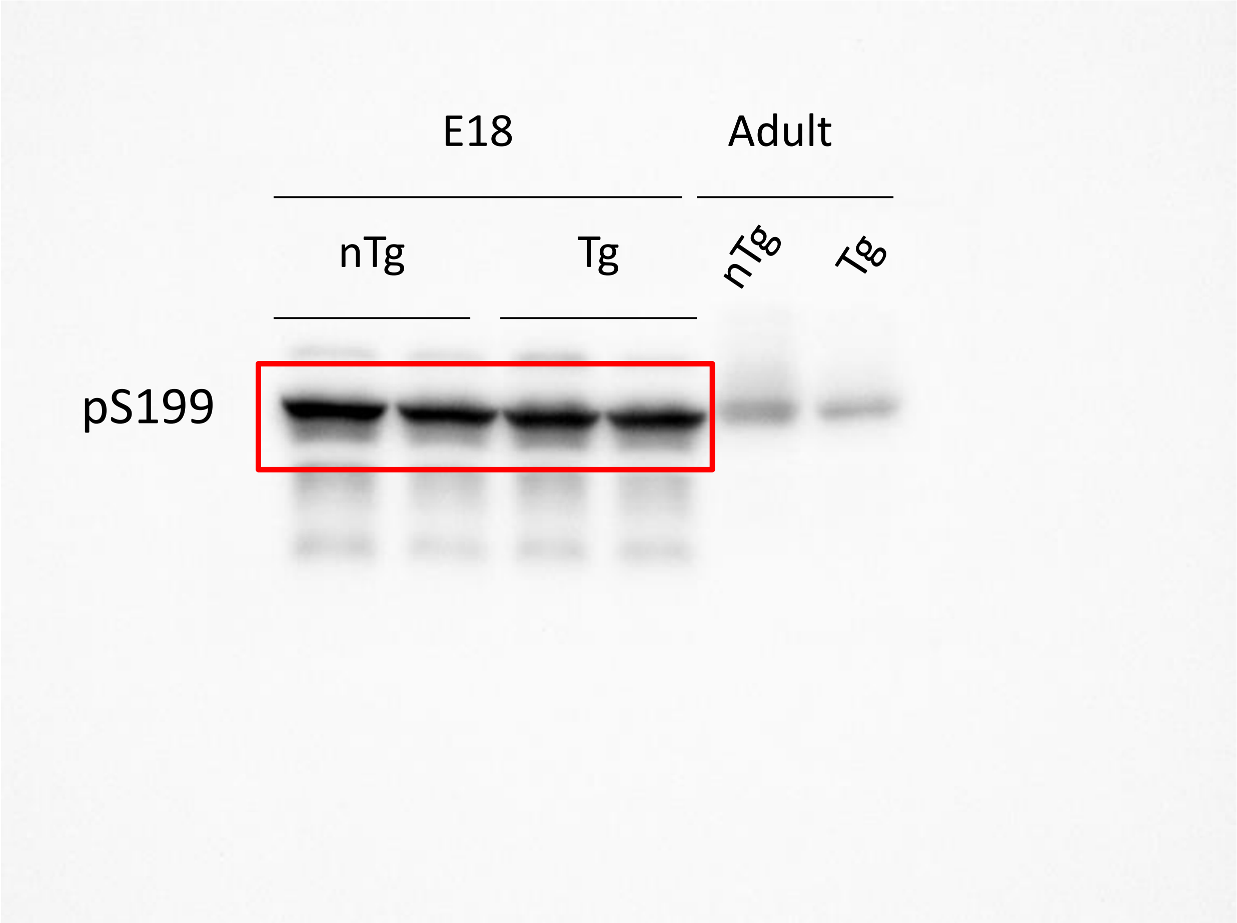

FIG 2B

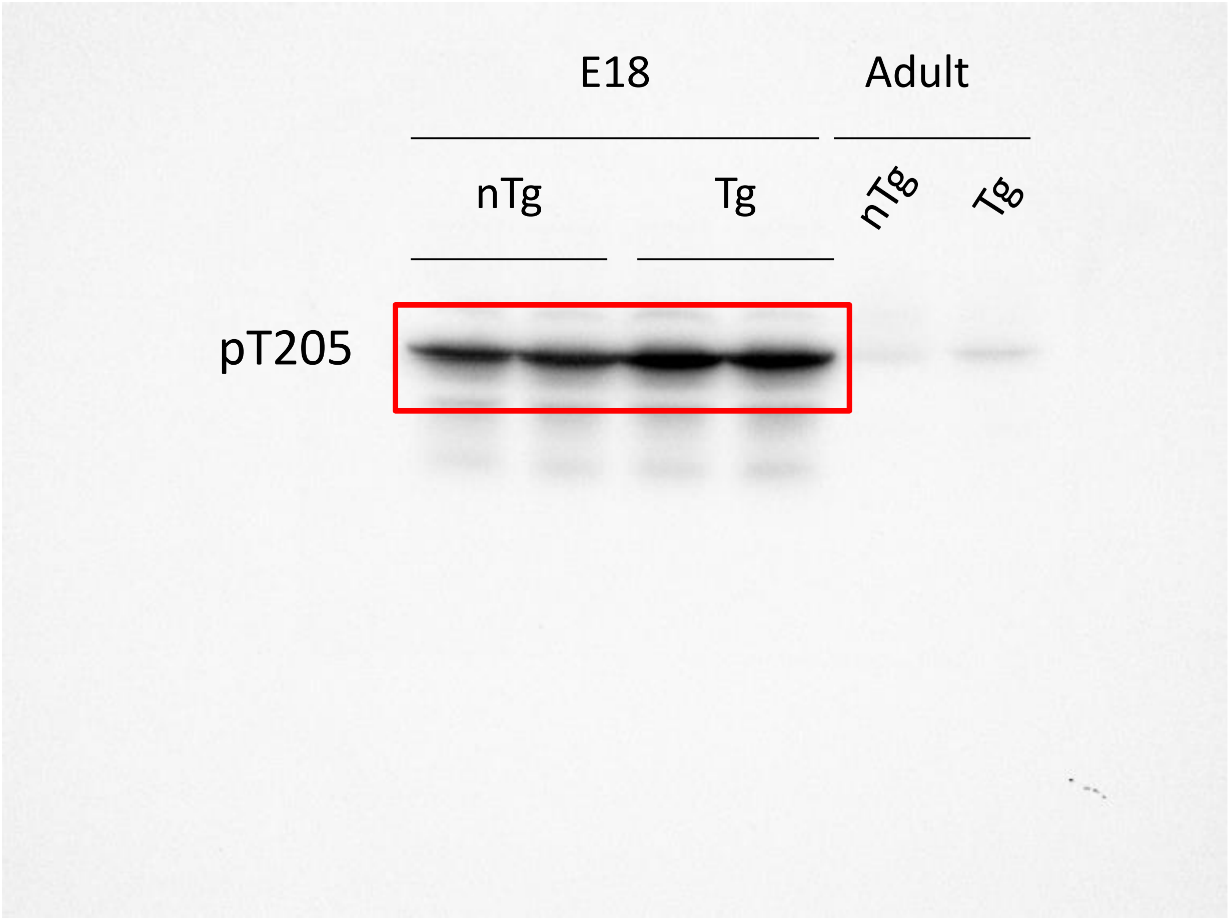

FIG 2B

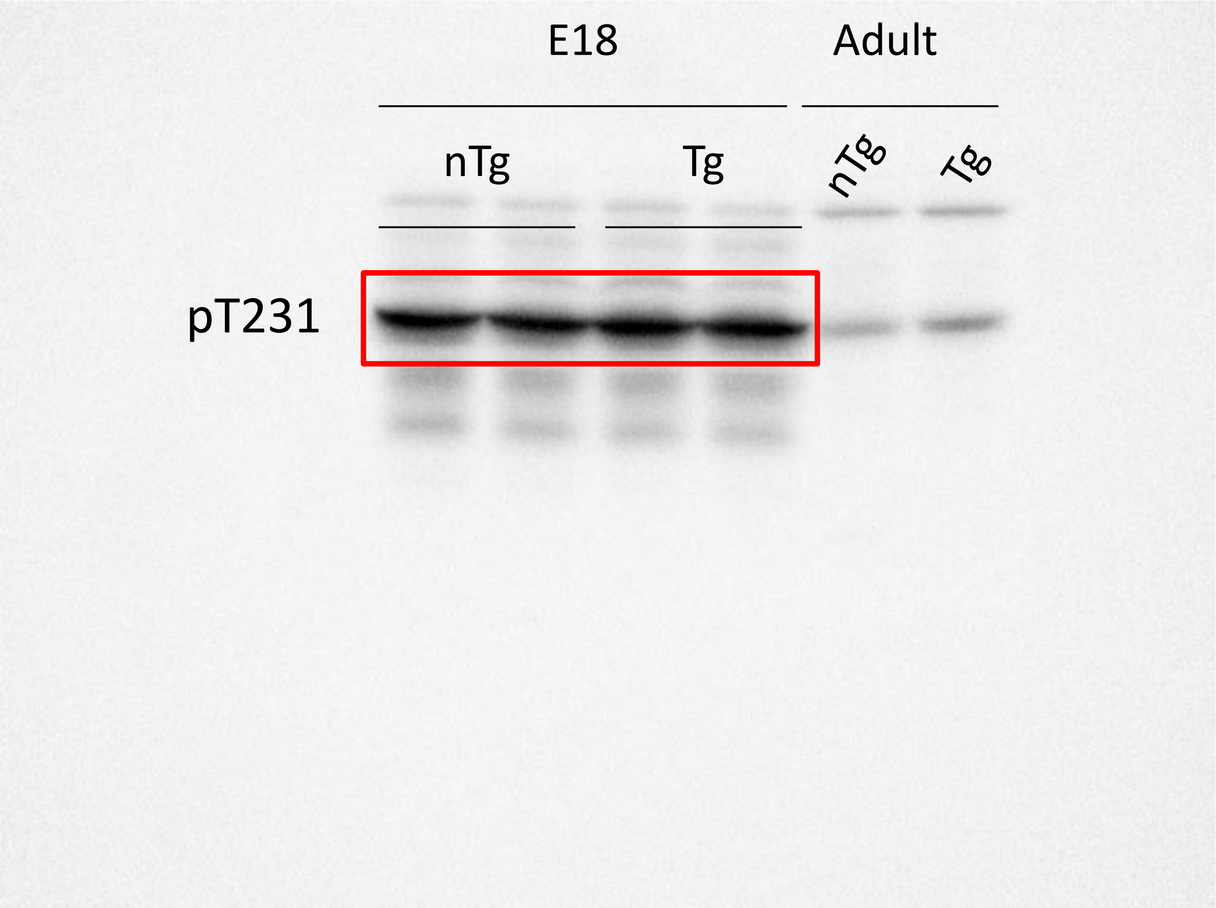

FIG 2B

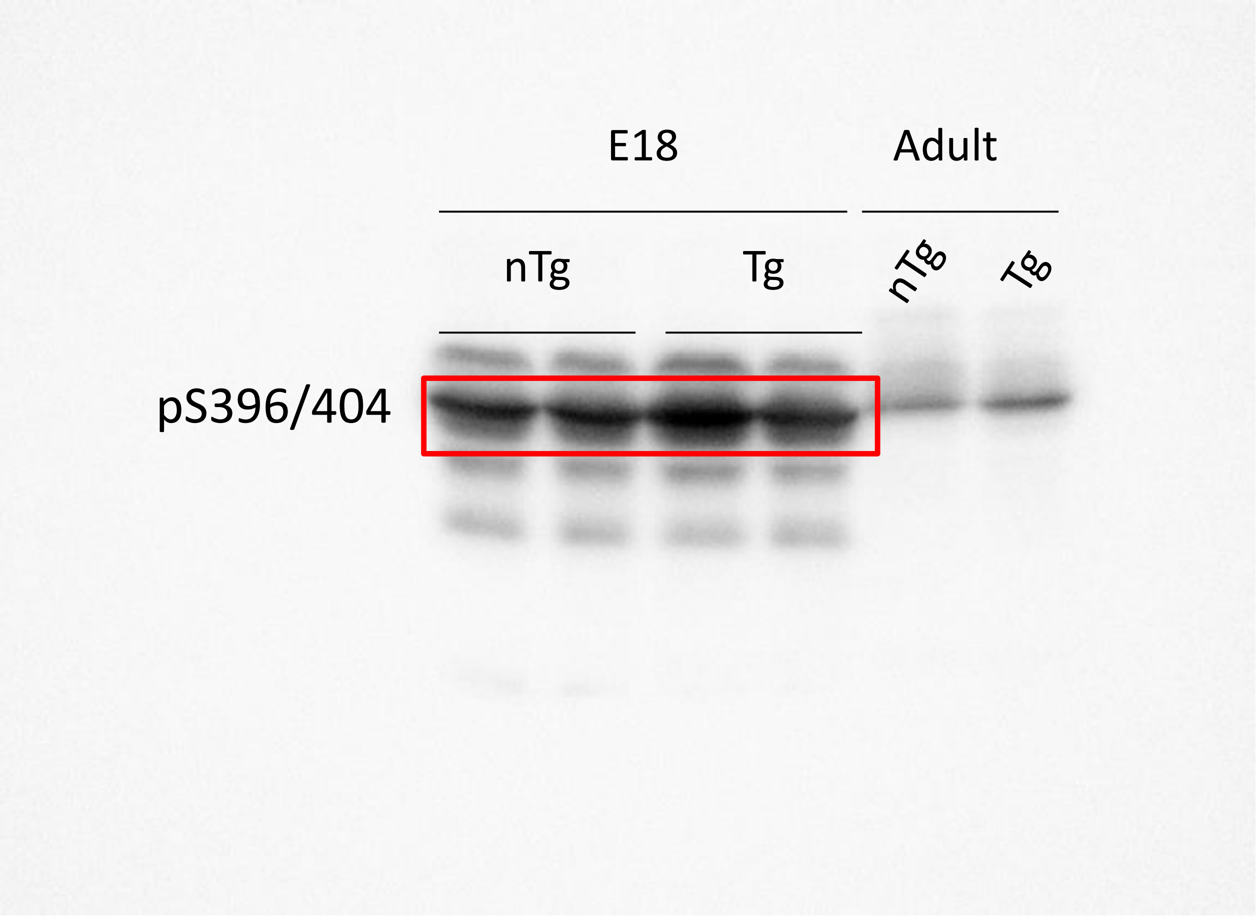

FIG 2B

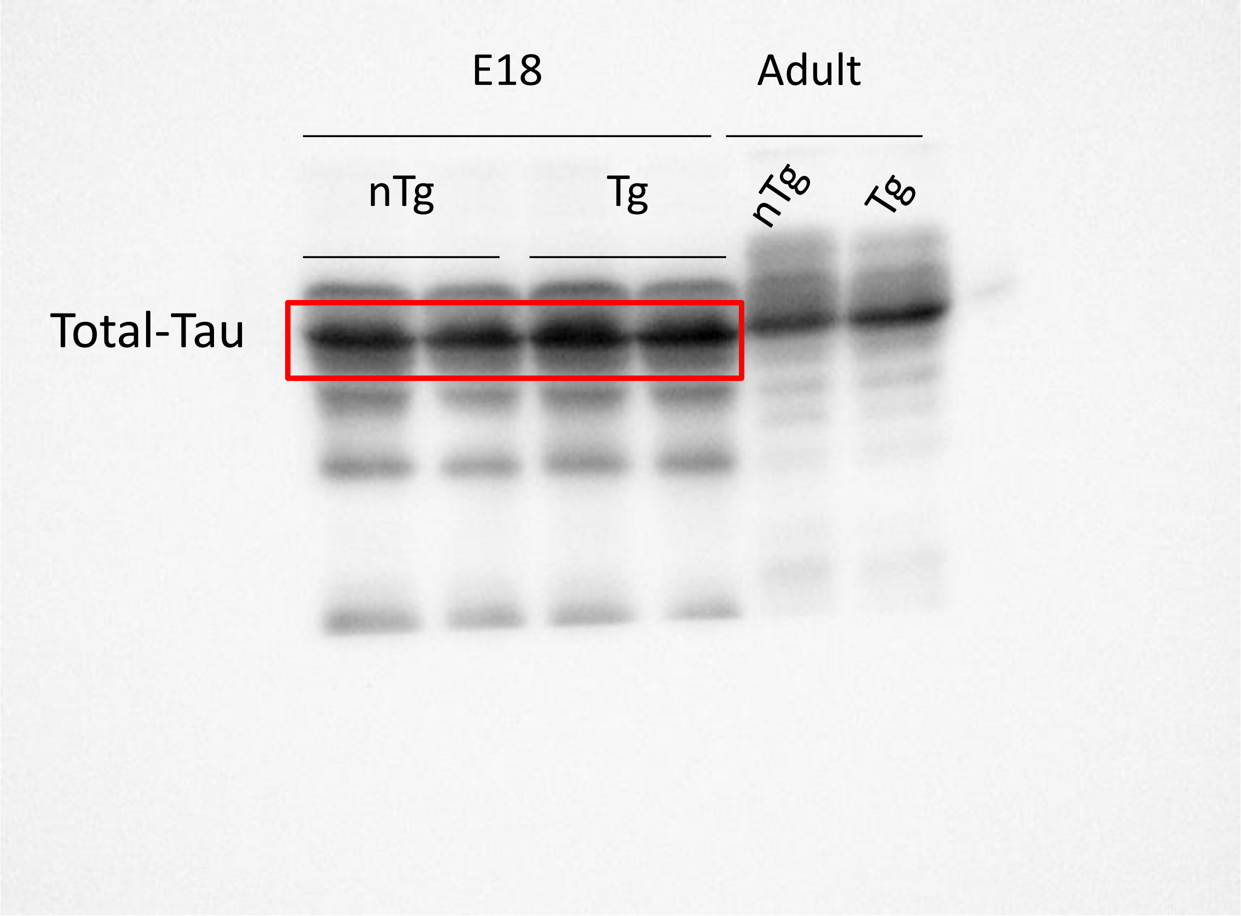

FIG 2B

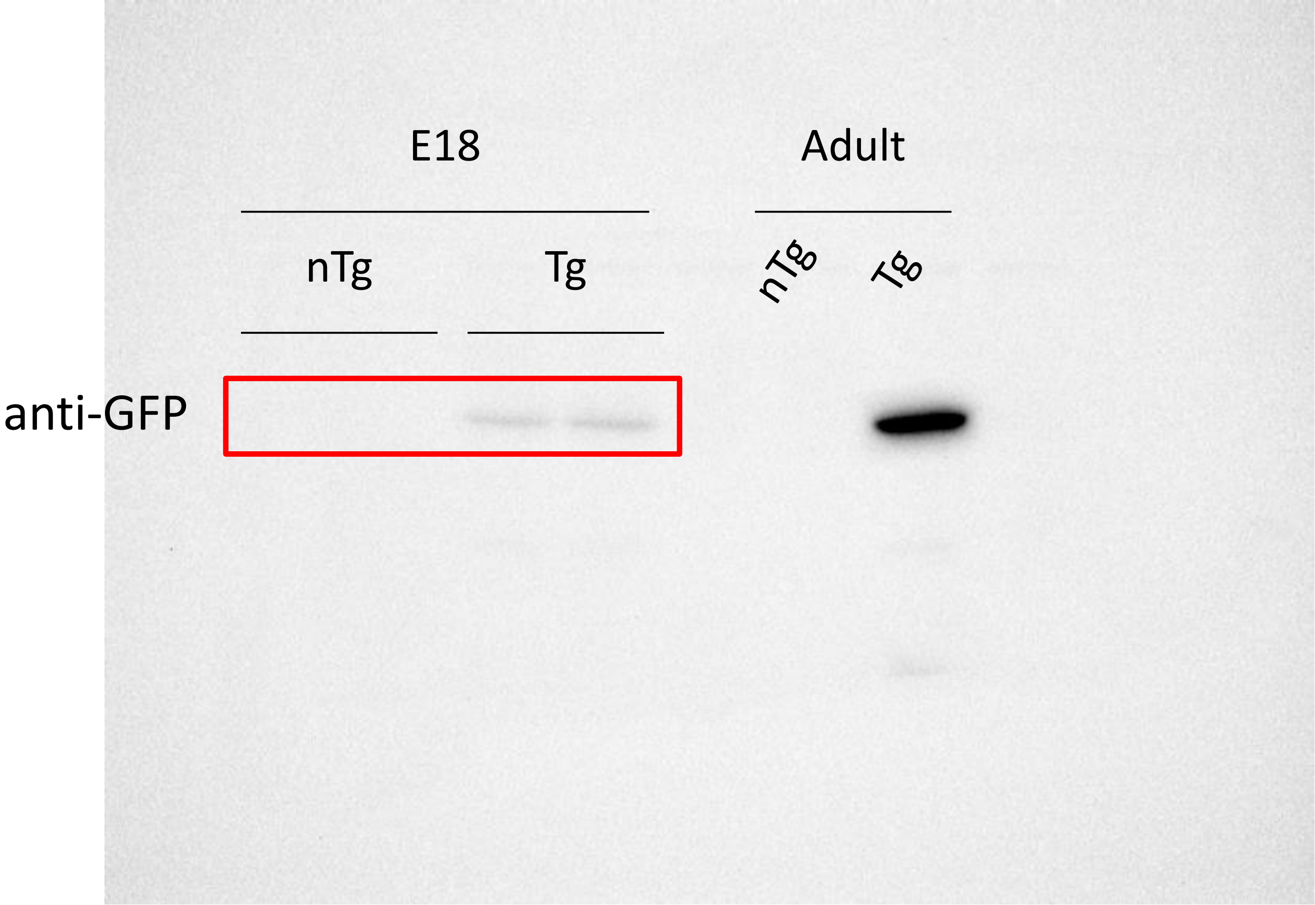

FIG 2B

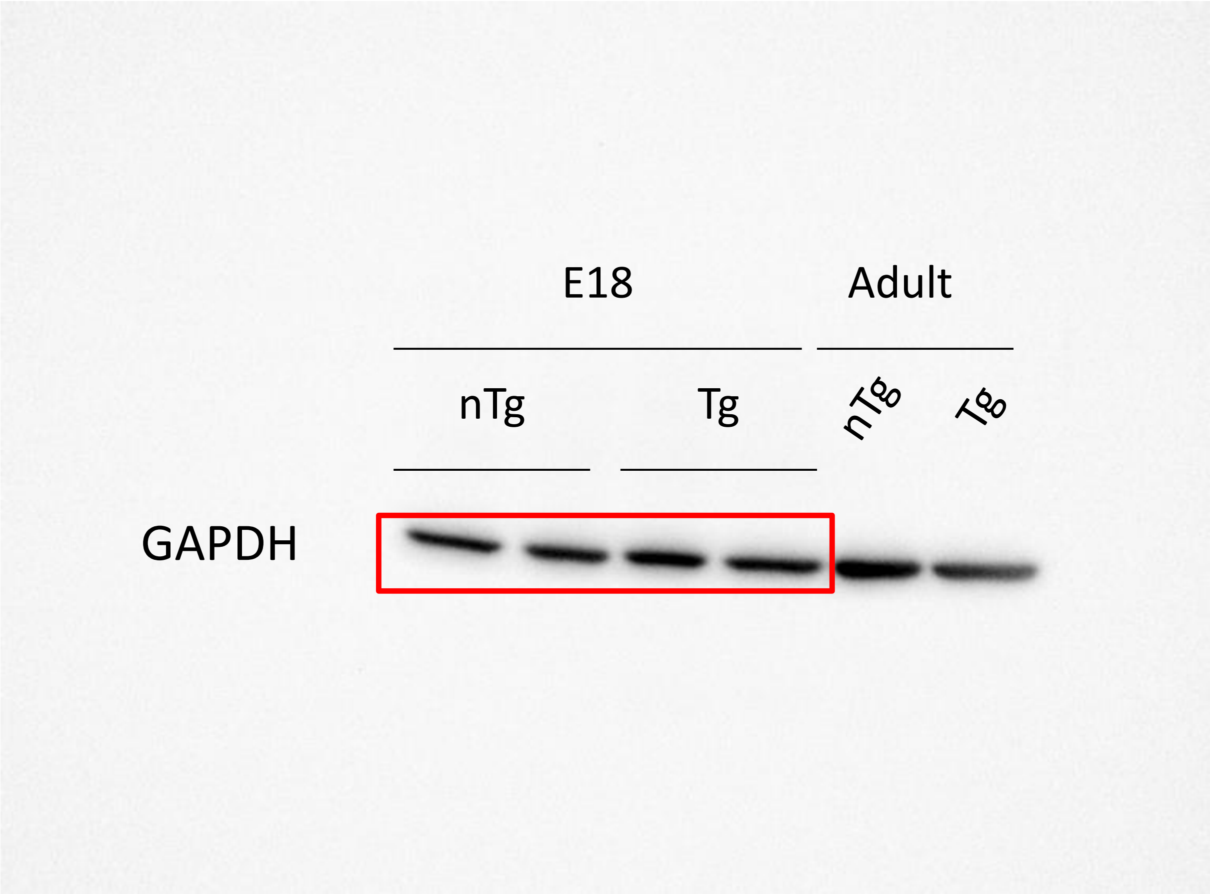

FIG 3A

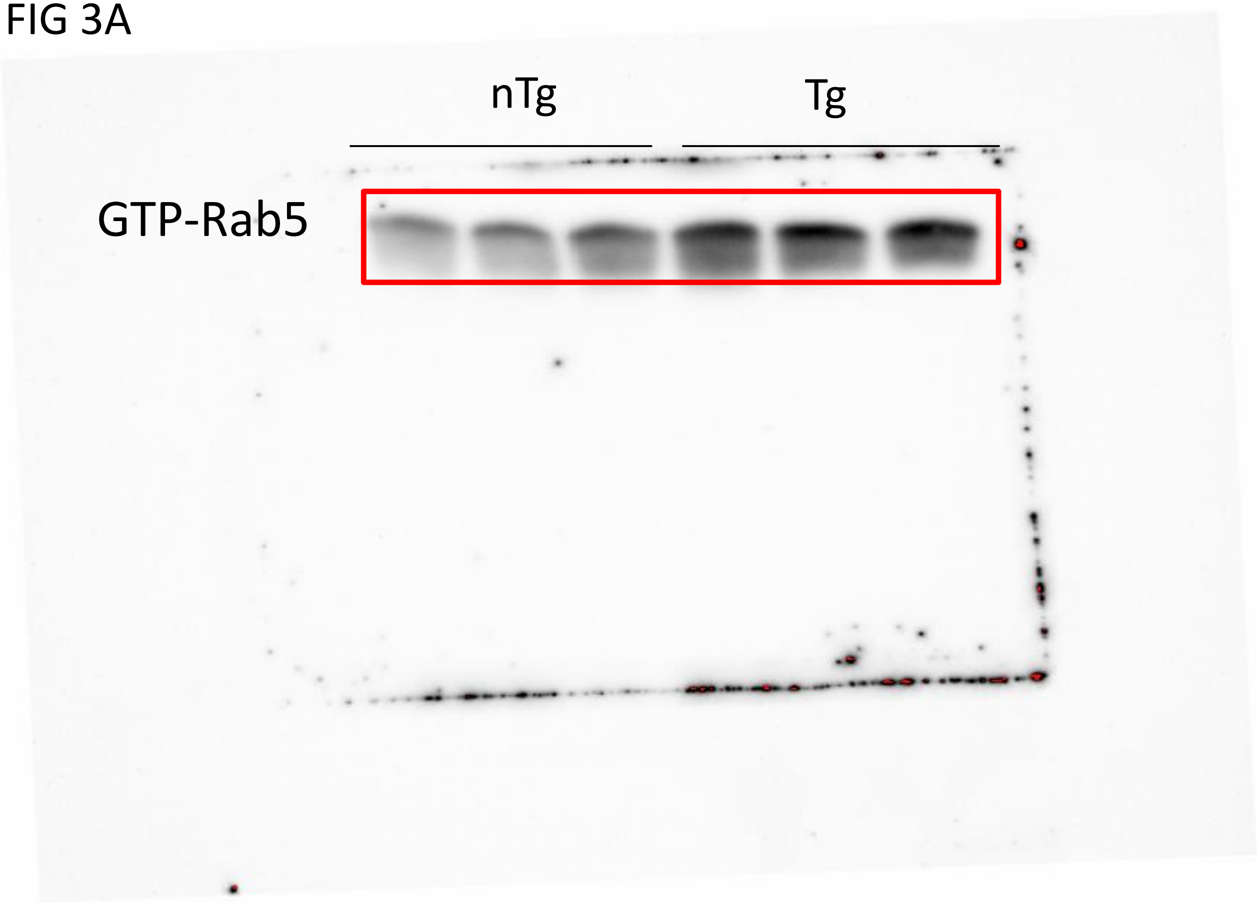

FIG 3A

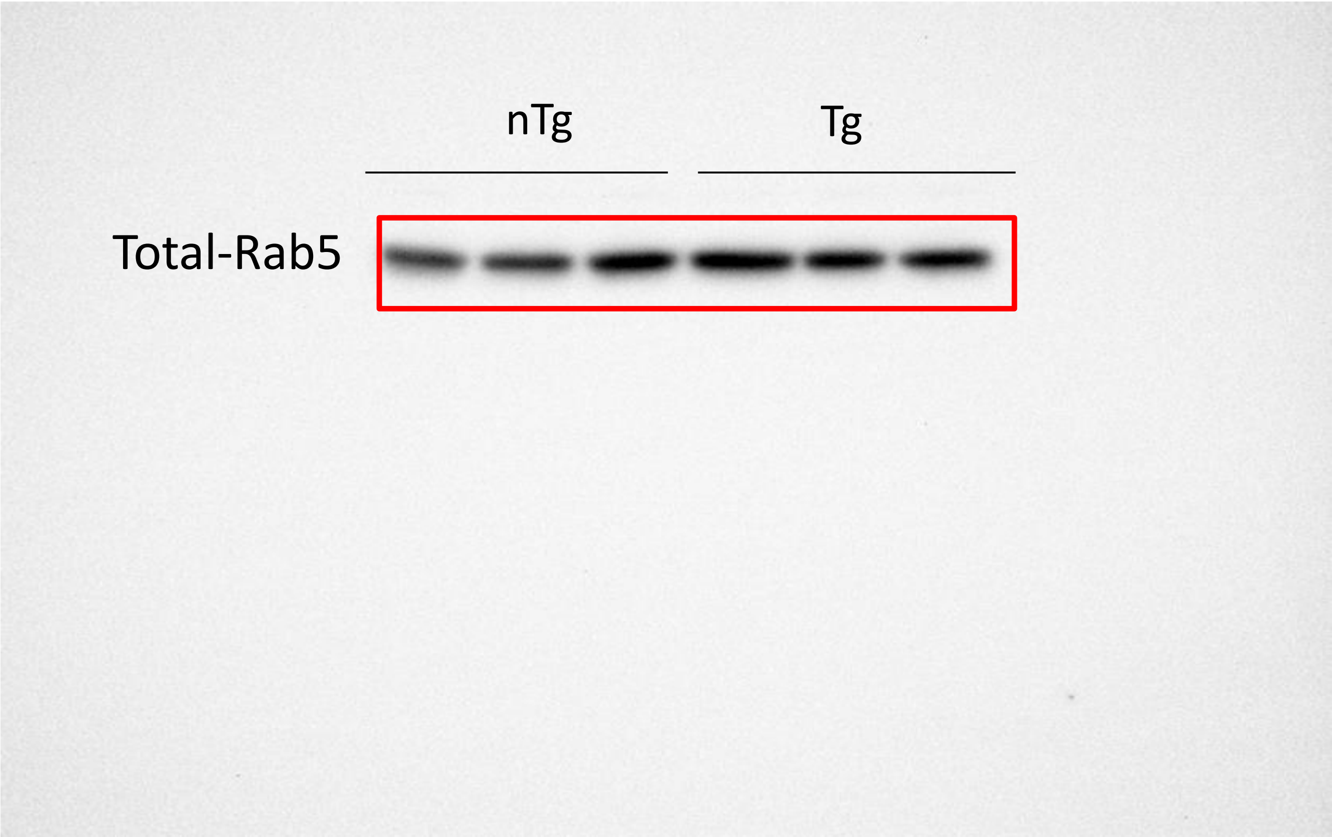

FIG 3A

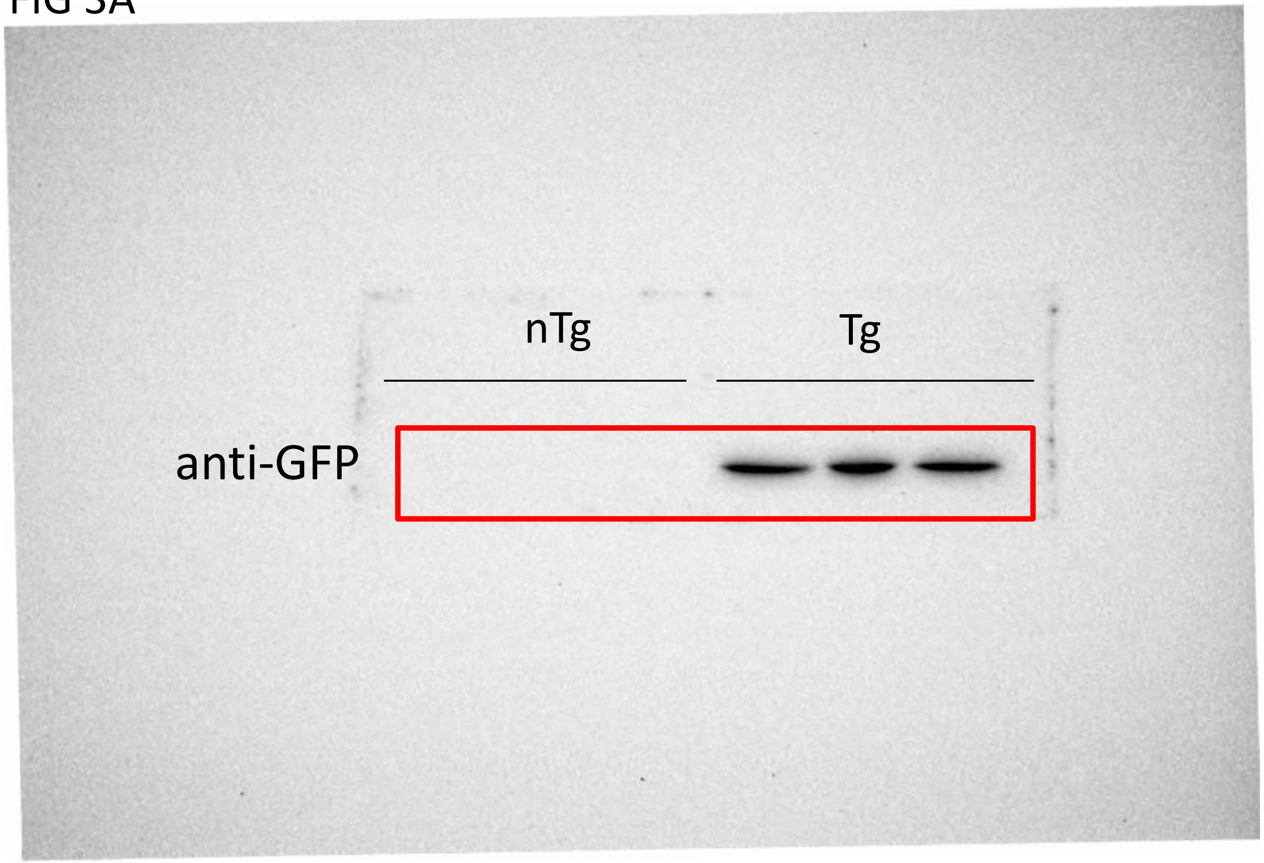

FIG 3A

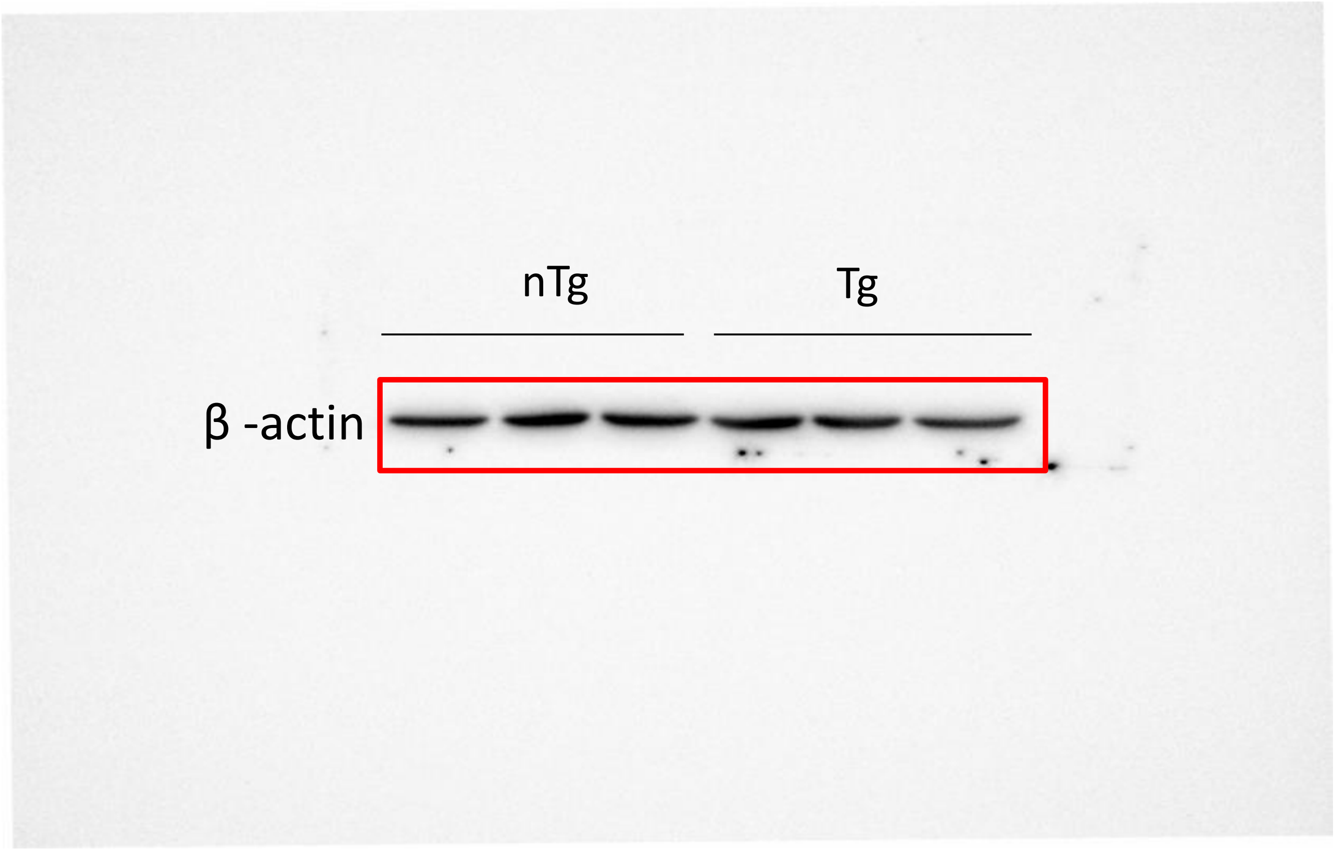

FIG 3C

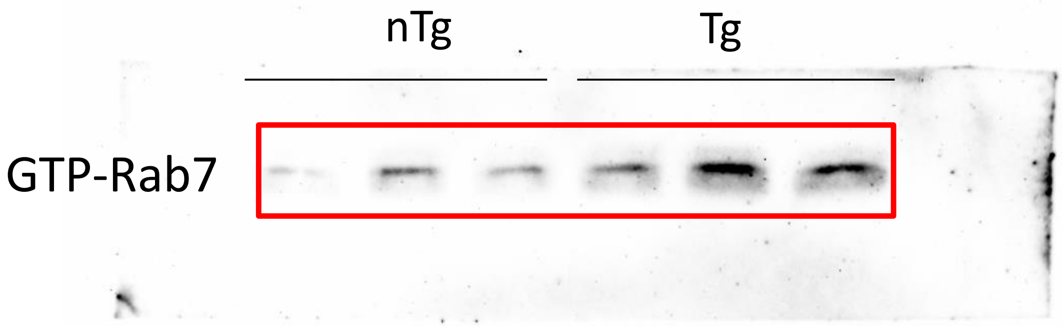

FIG 3C

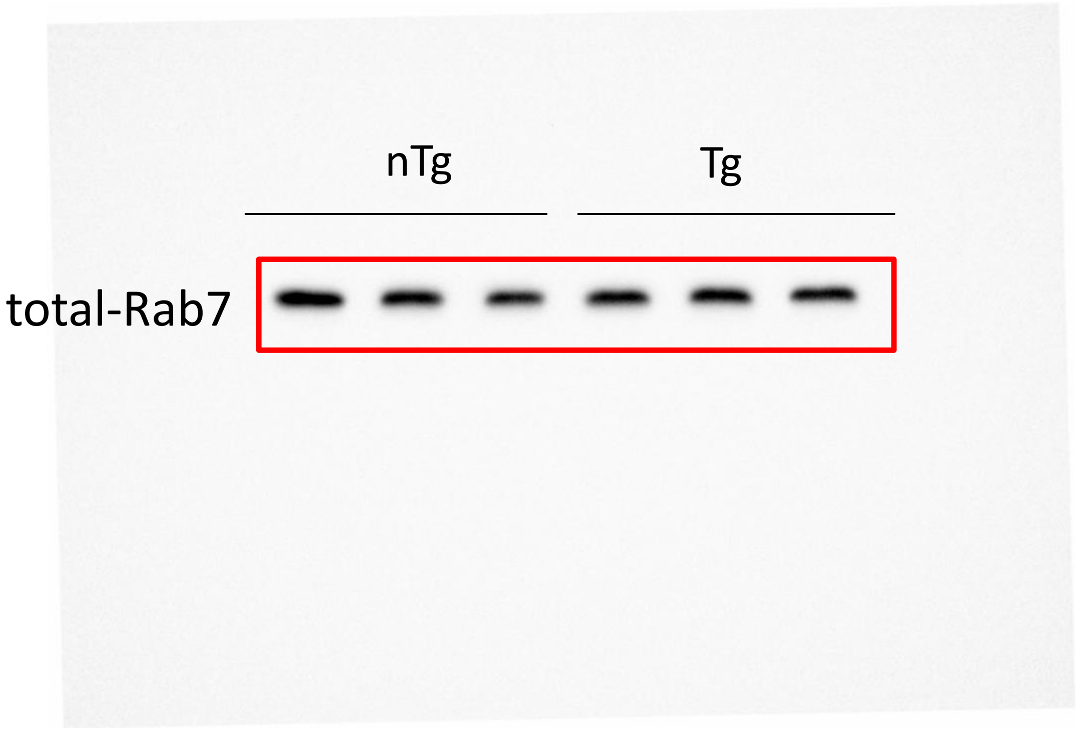

FIG 3C

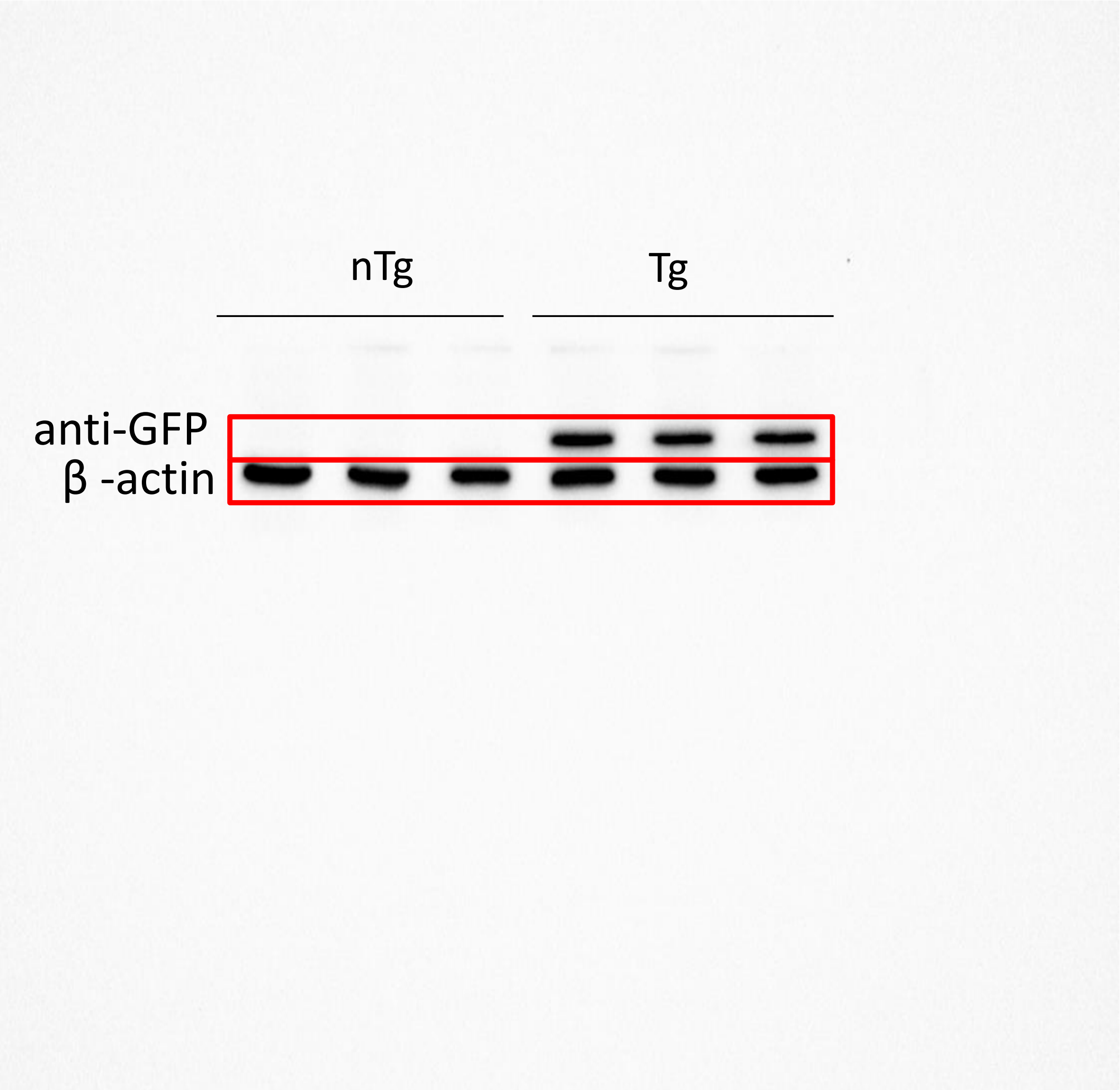

FIG 3E

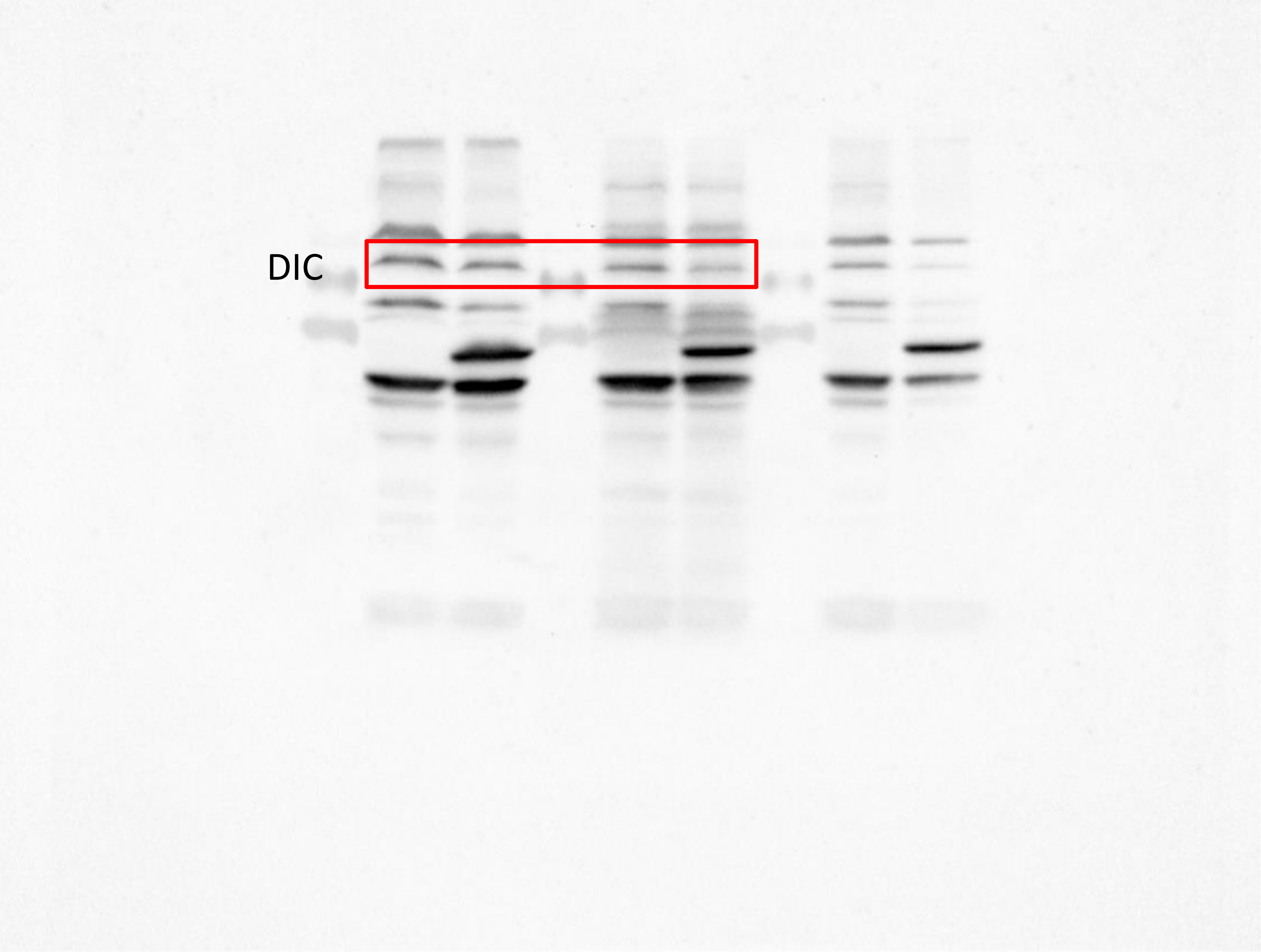

FIG 3E

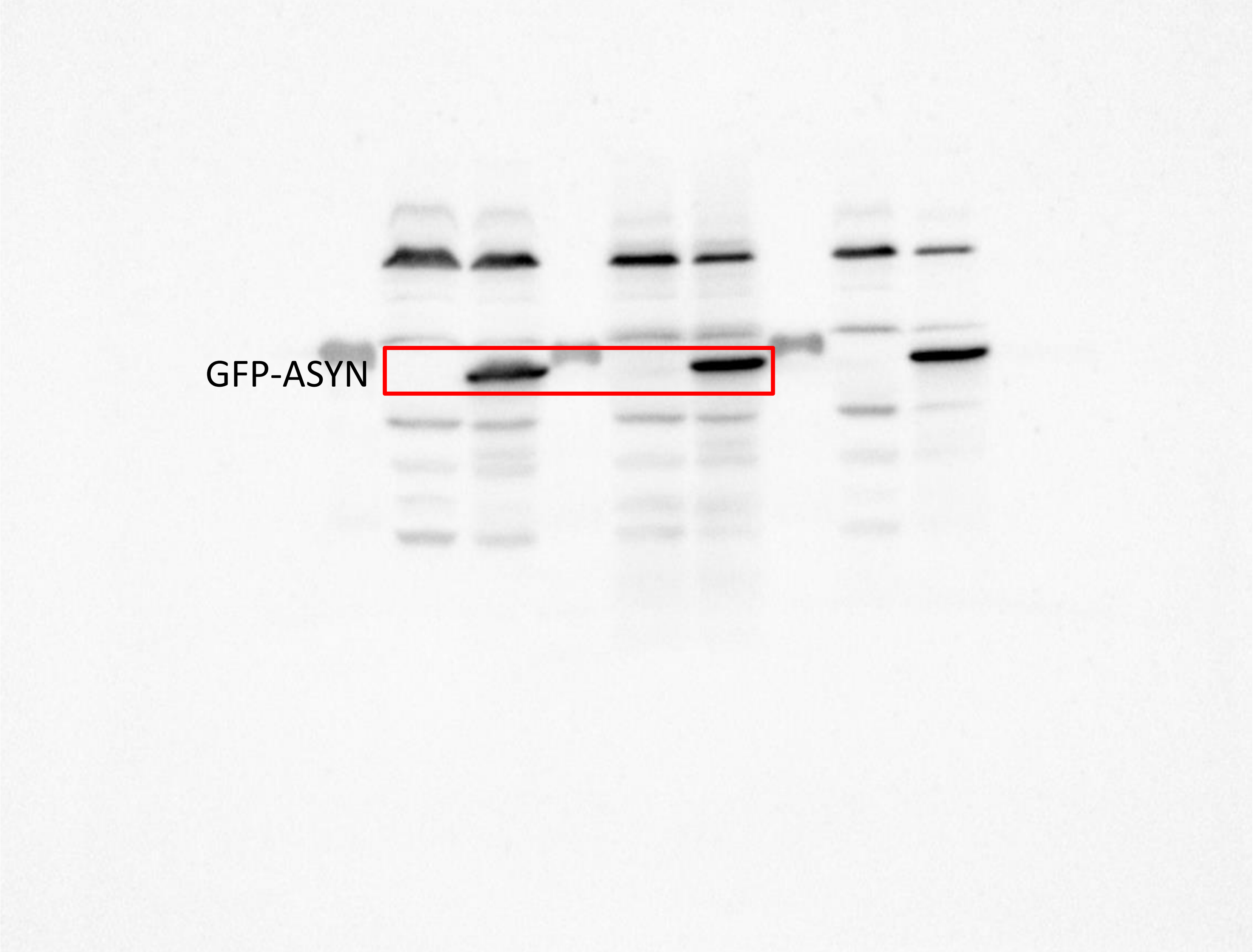

FIG 3E

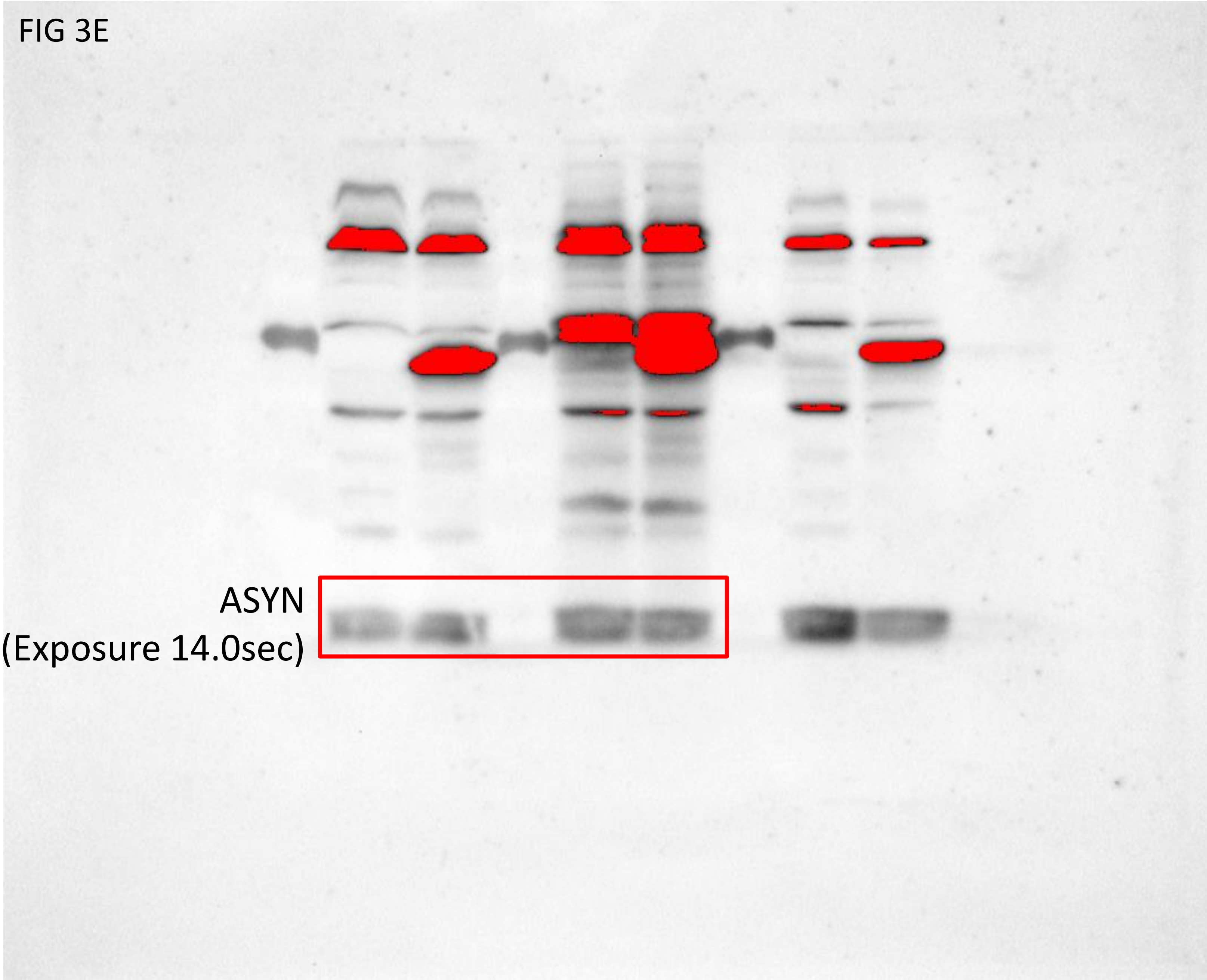

FIG 3E

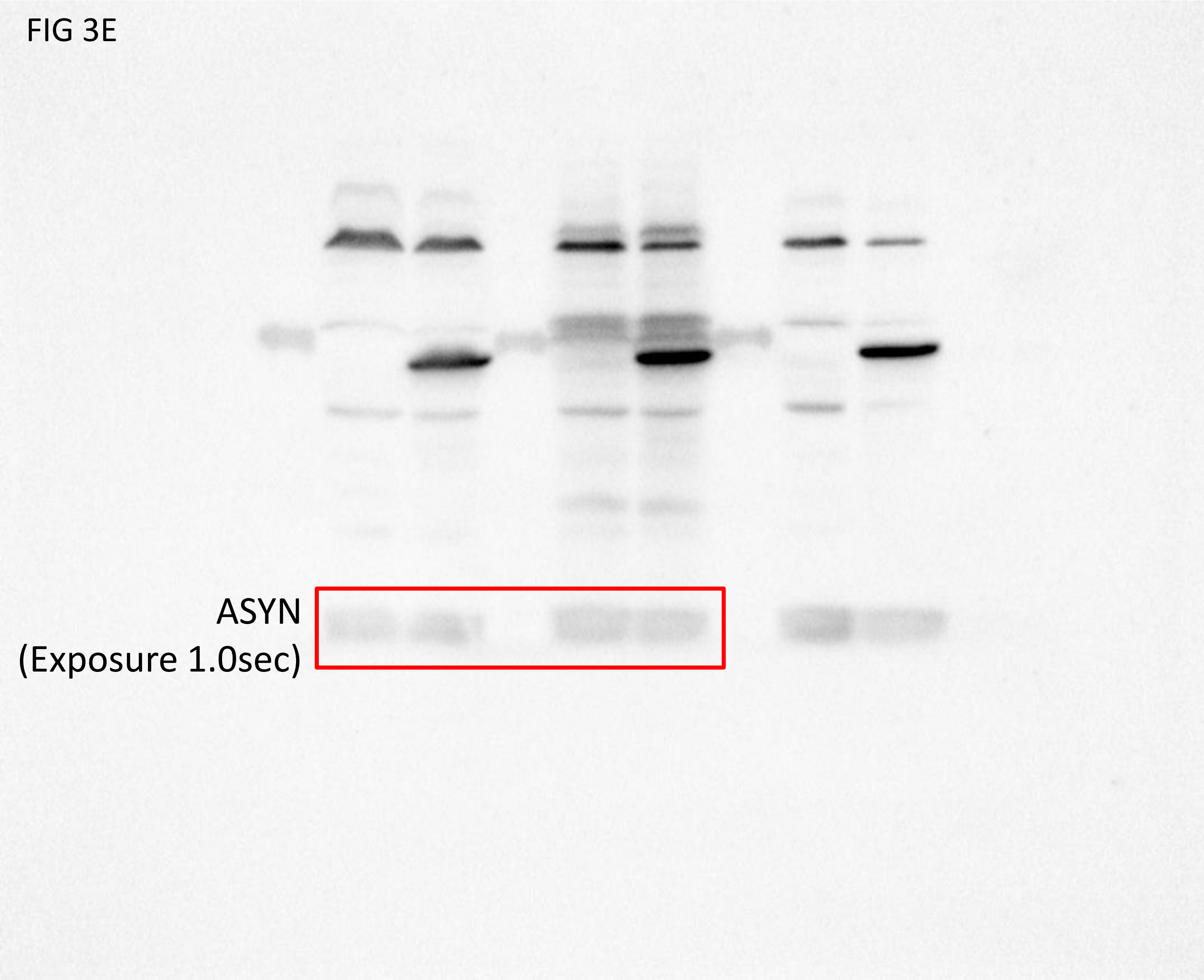

FIG 5A

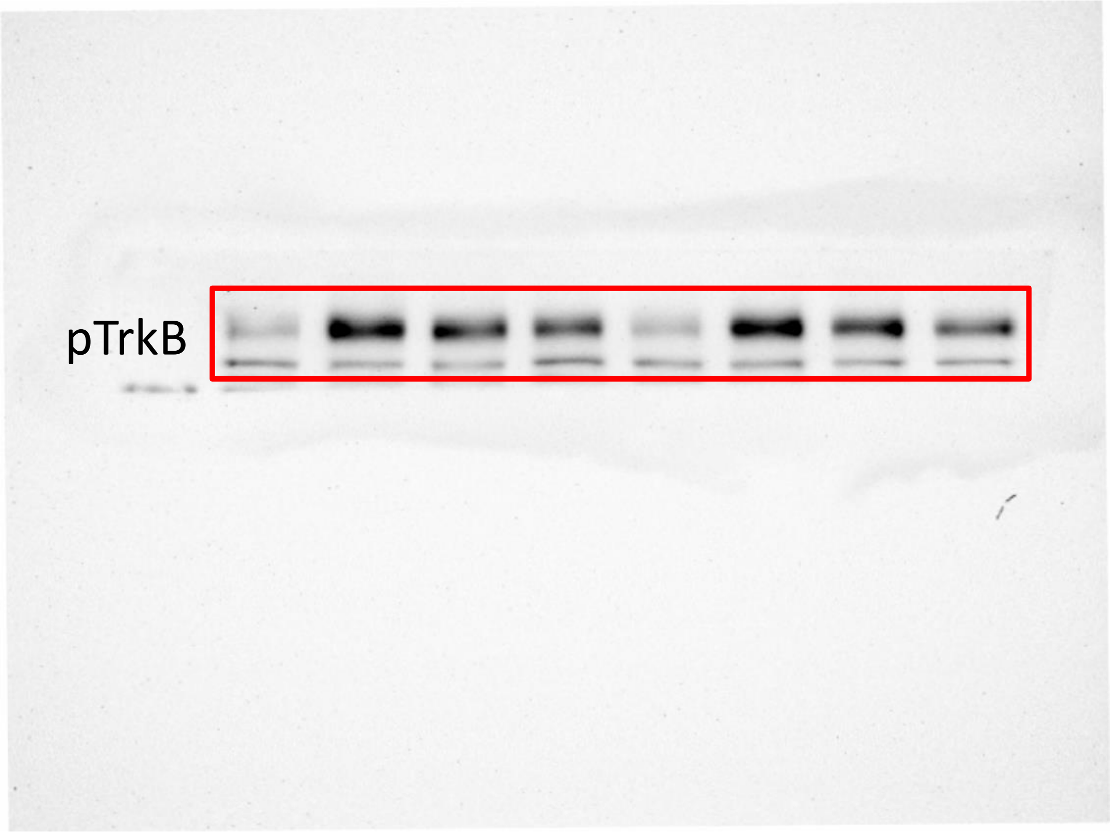

FIG 5A

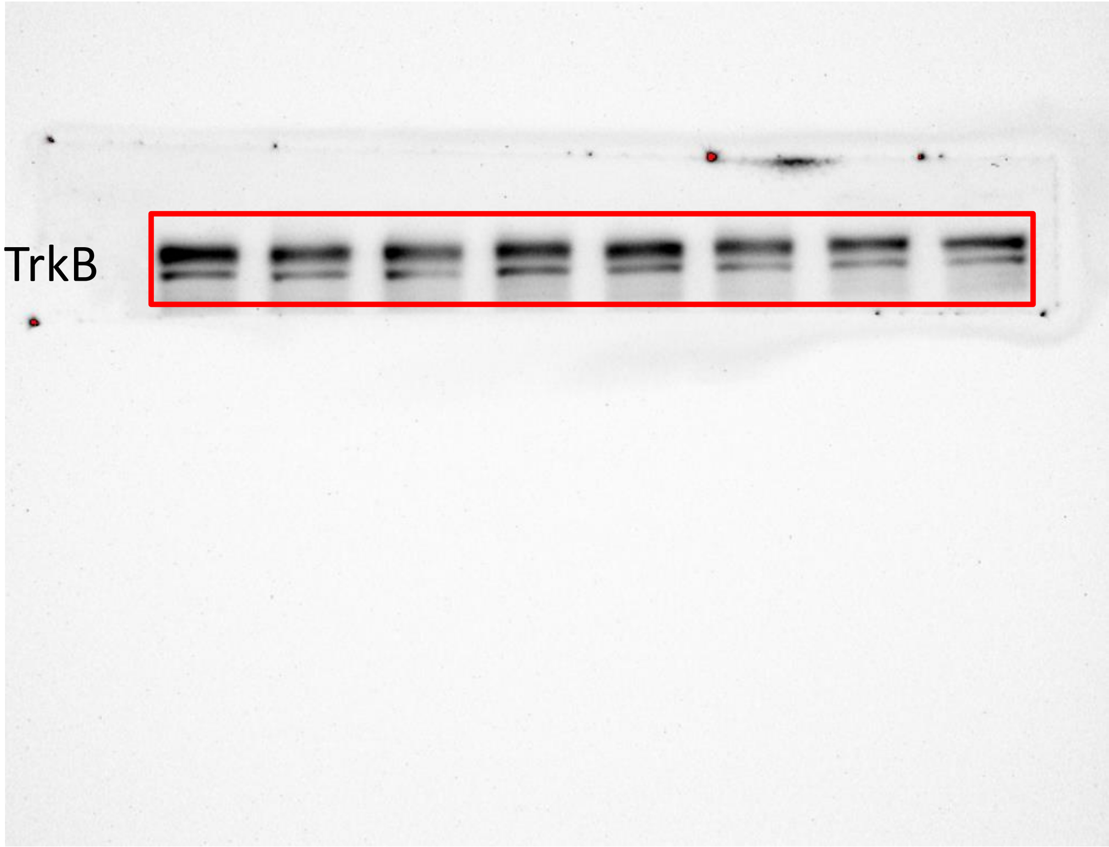

FIG 5A

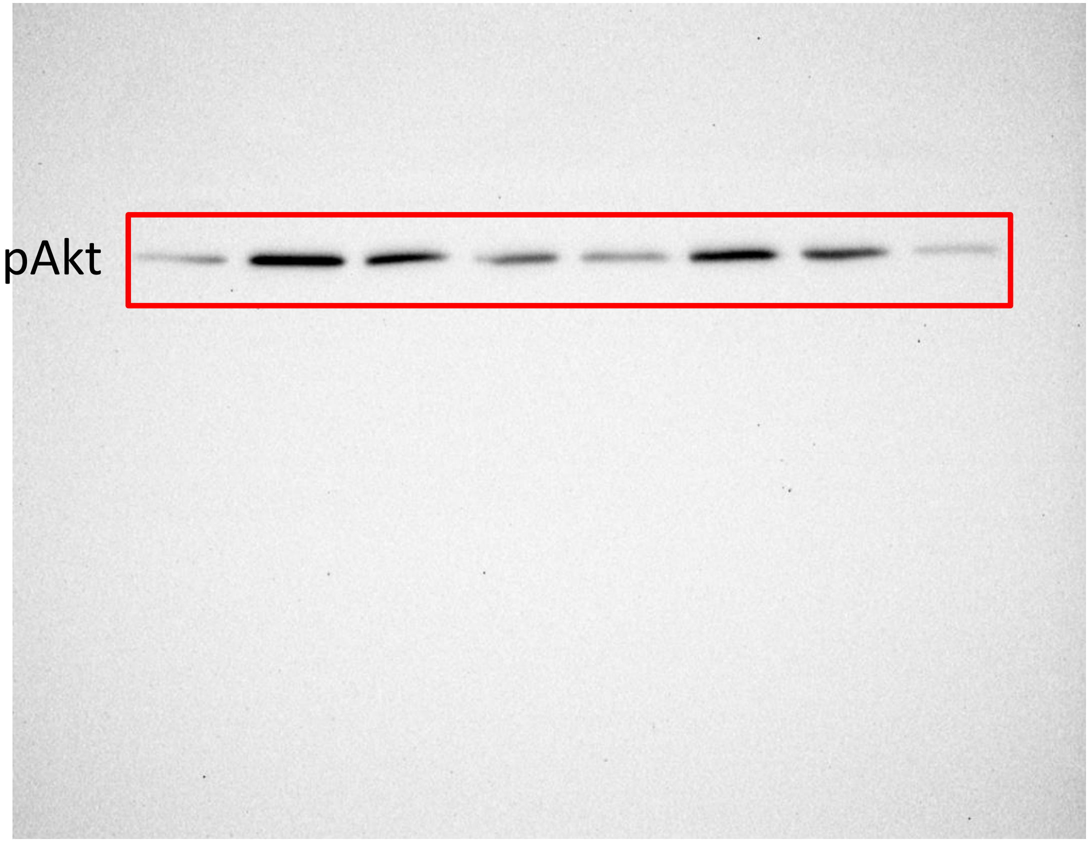

FIG 5A

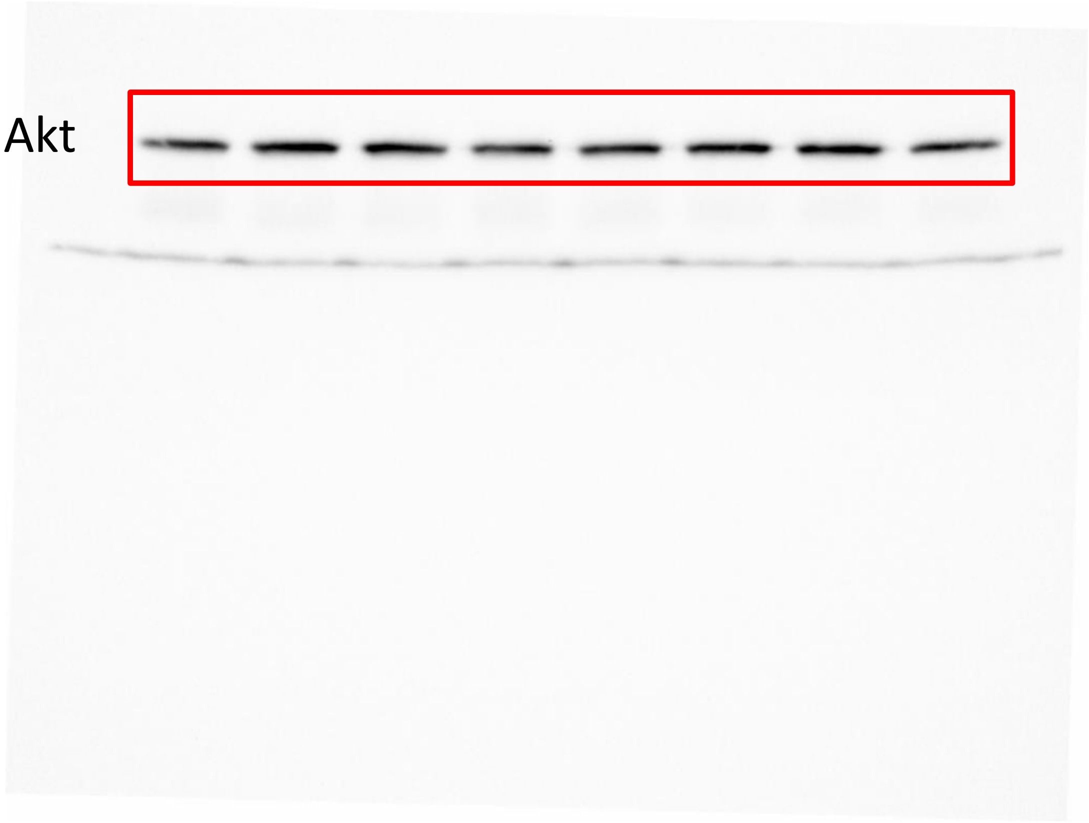

FIG 5A

pErk 1/2

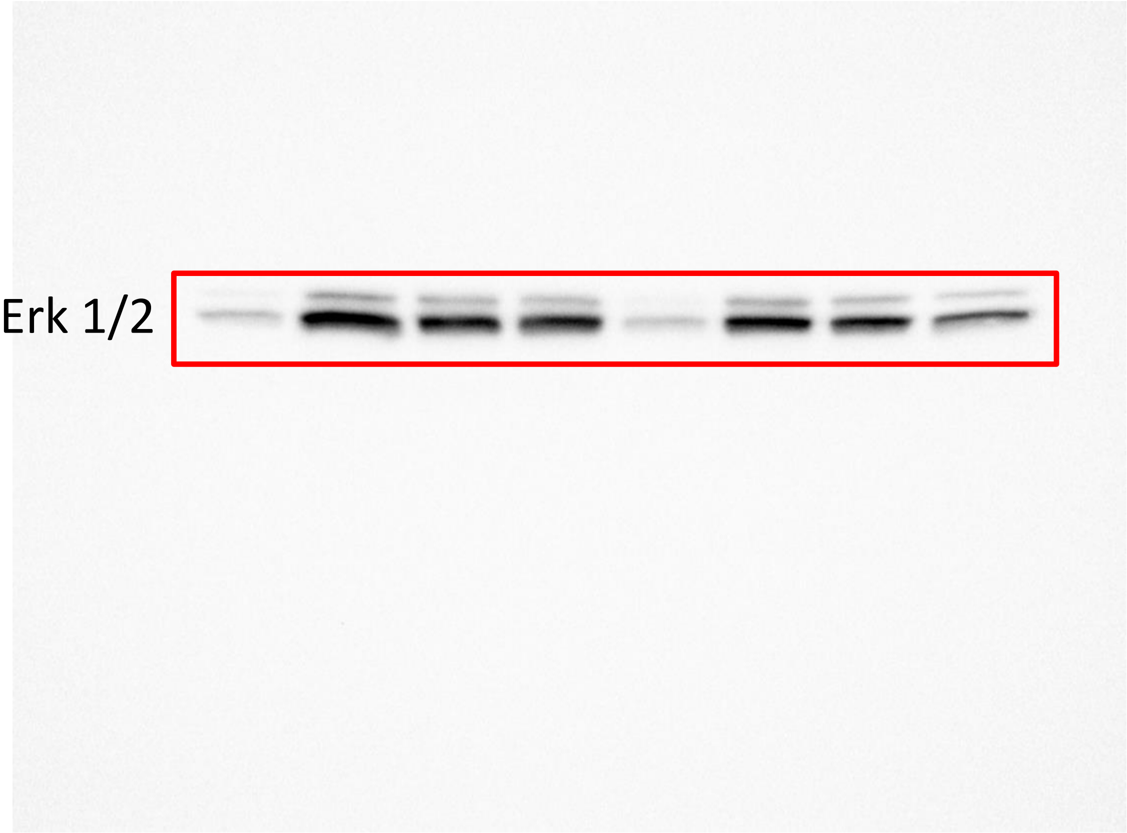

FIG 5A

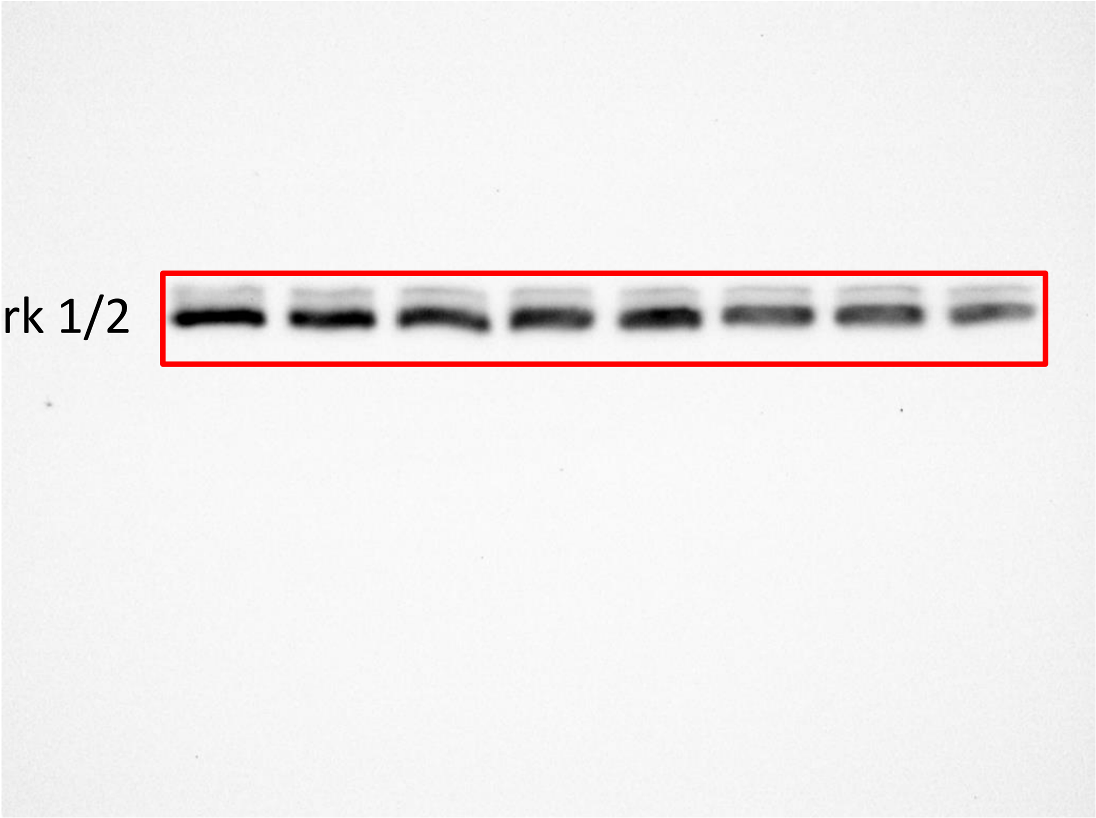

Erk 1/2

FIG 5A

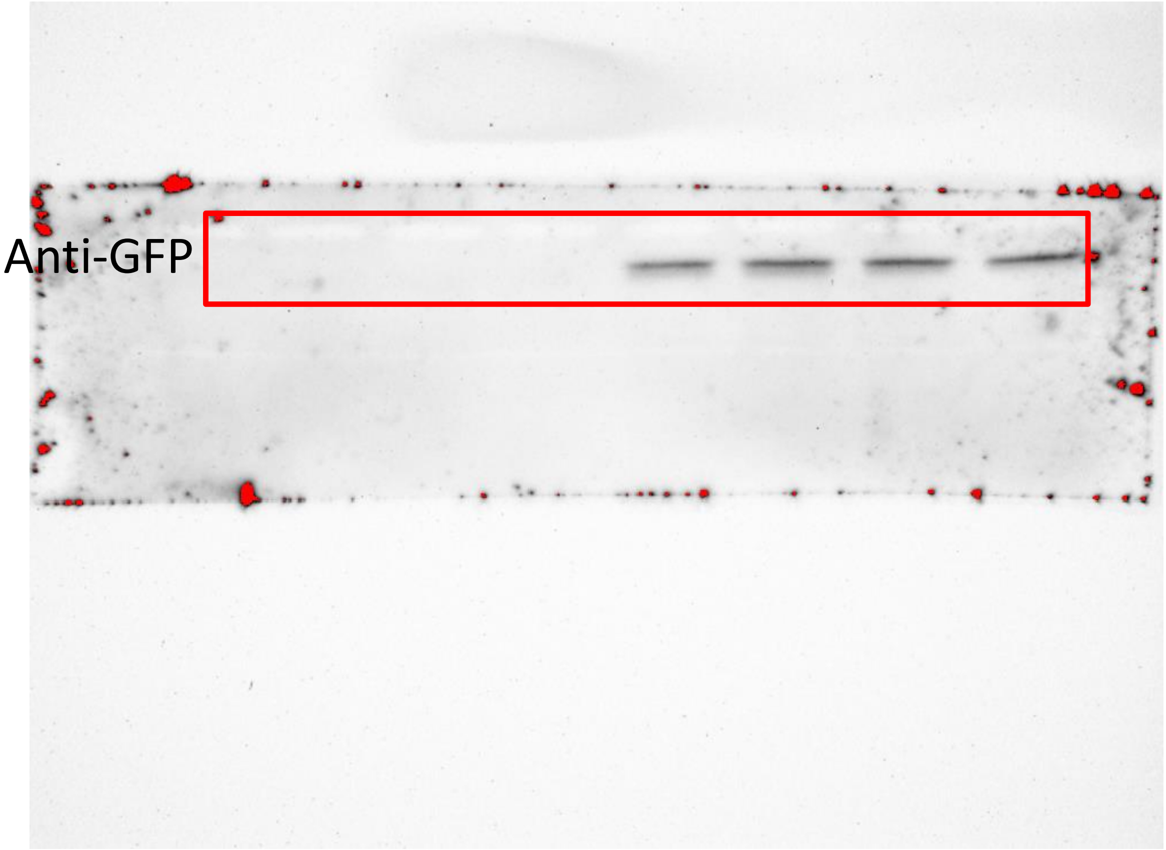

FIG 5A

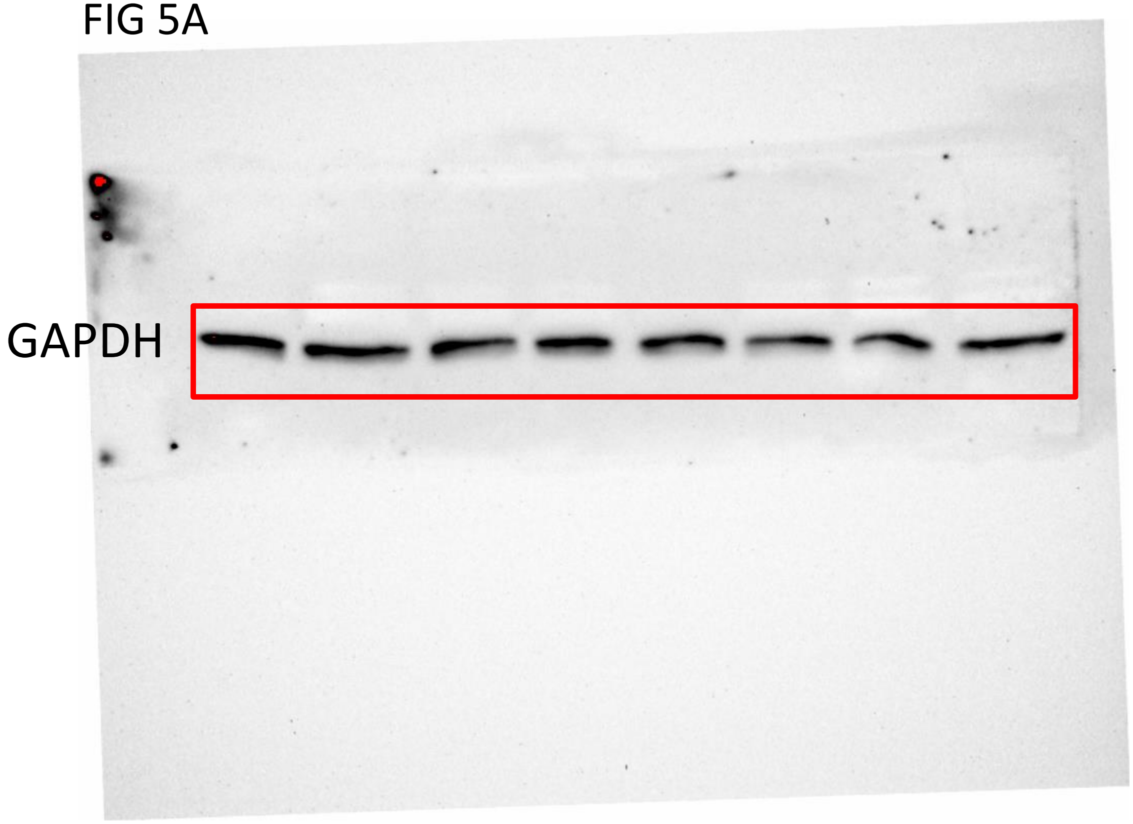

Supplement: Supplementary file 1 — Supplemental Information [file 41598_2017_4232_MOESM1_ESM.pdf]
